# The Current State-of-the-Art of the Processes Involved in the Chemical Recycling of Textile Waste

**DOI:** 10.3390/molecules30020299

**Published:** 2025-01-13

**Authors:** Urbain Nshokano Ndagano, Laura Cahill, Ciara Smullen, Jennifer Gaughran, Susan M. Kelleher

**Affiliations:** 1 School of Chemical Sciences, Dublin City University, D09 E432 Dublin, Ireland; urbain.ndagano@dcu.ie (U.N.N.);; 2 School of Physical Sciences, Dublin City University, D09 V209 Dublin, Ireland

**Keywords:** chemical recycling, depolymerisation, PET, nylon, elastane, textiles

## Abstract

The textile industry’s rapid growth and reliance on synthetic fibres have generated significant environmental pollution, highlighting the urgent need for sustainable waste management practices. Chemical recycling offers a promising pathway to reduce textile waste by converting used fibres into valuable raw materials, yet technical challenges remain due to the complex compositions of textile waste, such as dyes, additives, and blended fabrics.

## 1. Introduction

The textile industry is a major contributor to global environmental pollution [1,2]. According to the 2023 Preferred Fibre & Materials Market Report, there are over 100 million tonnes of textiles produced and consumed annually around the globe, with almost the entirety ending up in landfills or incineration at the end of their lifecycle [3]. This large quantity of textile waste contributes to the pollution of soil, water, and air sources. Chemicals and microfibres can leak into the surrounding environment and pollute waterways and soil, impacting all species of the ecosystem [4,5,6]. In 2022, the global fibre production increased again to a record 116 million tonnes and is expected to rise to 130 million tonnes by 2030 if the trend is maintained [3,7]. The share of recycled fibres decreased to 7.9% in 2022 from 8.5% in 2021, which was mainly due to a decrease in the market share of bottle-based polyester fibre. However, less than 1% of the global fibre market was produced from pre- and post-consumer recycled textiles in 2022 [3,8]. Thus, there is a need to reduce the use of fibres from virgin fossil-based materials and to invest in strategies that enable fibre and textile recycling to generate high-value new materials. Similarly, a new system for the textiles economy is needed in which clothes, fabric, and fibres re-enter the circular economy after use and never end up as waste. A system-level change approach which will capture the opportunities missed by the current linear textiles system is required.

Based on the production stage and use, textile waste is classified into three categories: pre-consumer, post-consumer, and industrial waste [9]. By definition, pre-consumer waste is generated during the manufacture of a product and consists of by-product materials from the textile, fibre, and cotton industries. Post-consumer waste consists of any type of garment or household article generated from manufactured textiles that the owner no longer needs and decides to discard. These articles are discarded because they are either worn out, damaged, outgrown, or out of style, and they are either given to charities, sold in second-hand shops, or disposed of into the trash and end up in municipal landfills [9]. In the European Union (EU), of the 5.8 million tonnes of textiles discarded by consumers every year, 1.5 million tonnes is recycled and a staggering 4.3 million tonnes is sent to landfill or burned for energy recovery through incineration.

### 1.1. Environmental Impact of Textile Waste

Since the 1990s, synthetic fibre production has overtaken cotton production and currently dominates the fibre market. In 2022, the textile industry produced around 76 million tonnes of synthetic fibres which represented approximately 65% of global fibre production [3,10]. Synthetic materials such as polyester, nylon, and acrylic, which are non-biodegradable fibres, can take up to 200 years to degrade in landfill [11]. To deal with such a large amount of textile waste, developed countries are shipping their waste to developing countries such as Chile and Kenya which then create large landfills that take up space and can spill into inhabited areas [12,13,14]. In Accra, Ghana, a 65-feet-high landfill site exists which largely consists of clothes [15,16]. This landfill presents an environmental hazard as chemicals and microfibres can leak into the surrounding environment and pollute waterways and soil, thereby impacting many species of the ecosystem. Globally, the textile industry is responsible for about 20% of clean water pollution from dyeing and finishing products [17]. In fact, a single laundry load of polyester clothing can release about 700,000 microplastic fibres that can end ultimately up in the food chain, and the majority of these microplastics are often released during the first few washes [17]. The presence of these fibres has been confirmed in human tissues; however, their effect has yet to be fully analysed and understood [18,19]. The attractive features of fast fashion, such as the ability of mass production at low prices, contribute to the manufacture of poorer-quality garments, leading to a larger release of microplastics between washes. In 2019, the European Environment Agency (EEA) published a report that showed that more than 500,000 kg of microplastics had been accumulated each year at the bottom of the oceans as the result of washing synthetic products [20]. Furthermore, the report also showed that pollution from garment manufacturing had a detrimental effect on animal, ecosystem, and human health in the area where the factories are located. Microplastics have been detected in teacups, human placenta, and blood, with their toxicity and hazardous effects being poorly known at this stage [21,22,23]. As part of its plan to achieve a circular economy by 2050, the EU aims to reduce the environmental impact of textile waste and increase the life cycle and recycling of textiles [24]. In 2023, the EEA published a report that showed that the textile industry was the third largest source of water degradation and land use in 2020 [25]. In that year, it took on average 9 m^3^ of water, 400 m^2^ of land, and 391 kg of raw materials to provide clothes and shoes for each EU citizen. Incineration is often used as a method to recover energy from textiles; however, harmful compounds such as carbon dioxide (CO_2_) and carbon monoxide are generated during the process [26,27]. In comparison to landfill, incineration is the preferable method, as the energy recovered can be used in place of fossil fuels. Thus, the textile recycling process could provide an avenue for sustainable textile waste and the production of new textiles that do not originate from virgin fibres or fossil fuel materials.

A successful recycling process should be more sustainable than the current end-of-life options for textiles and less harmful to the environment than the process of producing new textiles from virgin fibres. Currently, there is very little information about the environmental impact of textile recycling processes; hence, energy consumption is the primary measure of the environmental impact of the process. Laboratory experiments are carried out to measure the energy cost in kilowatt hour (kWh) of each phase of the process that requires the use of electricity. In addition, the carbon footprint (CF), which is the amount of greenhouse gas (GHG) emissions generated by the activity in kg CO_2_-equivalent, of the recycling process can also be used to determine the carbon footprint of the chemical recycling of textiles [28]. Typically, the CF of textiles depends on the type of fabrics and production processes. For instance, natural fibres such as cotton and silk have a lower CF than synthetic fabrics such as polyester and nylon, which require more energy and chemicals during the manufacturing process. Furthermore, fabrics manufactured through eco-friendly processes such as closed-loop systems have a significantly lower CF than those produced using conventional methods. The energy cost and the CF can be used to establish how many kilograms of CO_2_ are released per kWh of energy, also known as carbon intensity. Carbon intensity varies based on the sustainability of the fuel source, with renewable sources such as solar, wind, and hydro power producing minimal carbon emissions compared to fossil fuels [29,30]. The textile industry is estimated to be responsible for 10% of the global carbon emissions, which is more than the combined emissions generated by maritime shipping and international flights [31]. The 2023 EEA report also showed that textile purchases in the EU in 2020 generated about 270 kg of CO_2_ emissions per person [32]. In other words, from the textile products consumed in the EU, 121 million tonnes of GHG were generated. Li et al.’s case study of jeans showed that the CF of fast fashion consumption is 2.50 kg of CO_2_ equivalent per one wear jeans, which is 11 times higher than that of traditional fashion consumption [33]. In addition, the manufacturing and transportation of jeans accounted for 91% of the CF of fast fashion consumption. Leal Filho et al. highlighted the need to reduce the CF of the textile sector by proposing the utilisation of sustainable materials, investing in energy-efficiency technologies, reducing waste and water usage, and streamlining logistics [34].

### 1.2. Why Recycle?

**Figure 1:**
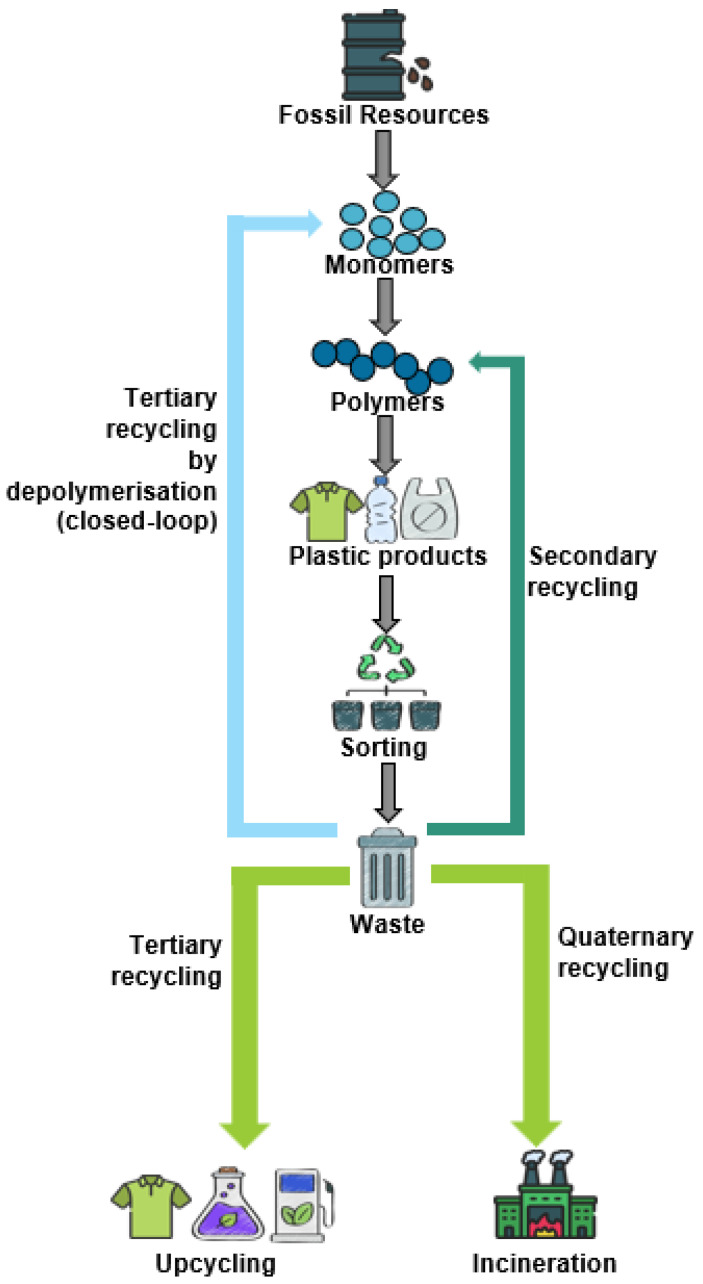
A schematic showing the stages of plastics and textile recycling [35].

Textile recycling is the process of repurposing used clothing and textiles to reprocess them into new products. It is seen as a sustainable technology for environmental conservation as it enables resource conservation, energy efficiency, and pollution reduction. However, textile recycling is not a uniform process, as it is dependent on the type of fabric used. Thus, each type of fabric has a distinctive recycling methodology. An example of a plastics and textile recycling process involving three stages is shown in Figure 1 [35]. The first stage involves the collection, processing, sorting, and cleaning of the old textile products so that they are ready for the recycling or manufacturing of new products. The second stage involves the manufacturing of new products from the raw material obtained by the processing of the old products. Pre-consumer textile materials can either be recycled mechanically, in which fibres are pulled apart and reworked into yarn, or chemically, where fibres are repolymerised into a chemical and spun. The third stage involves the purchasing of recycled goods by consumers directly from the production plants, thereby completing the recycling loop. The advantage of recycling is that the process is cost-effective, enables job creation, and contributes to the protection of the environment. The low water and energy consumption also make recycling much cheaper than manufacturing new textile products from raw materials. Recycling is a financially rewarding process, with people receiving money for turning in certain recyclable products [9,36], produces less greenhouse gases due to industries burning fewer fossil fuels [37], and provides opportunities for small and family businesses [9,38].

### 1.3. Textile Recycling Classifications

**Figure 2:**
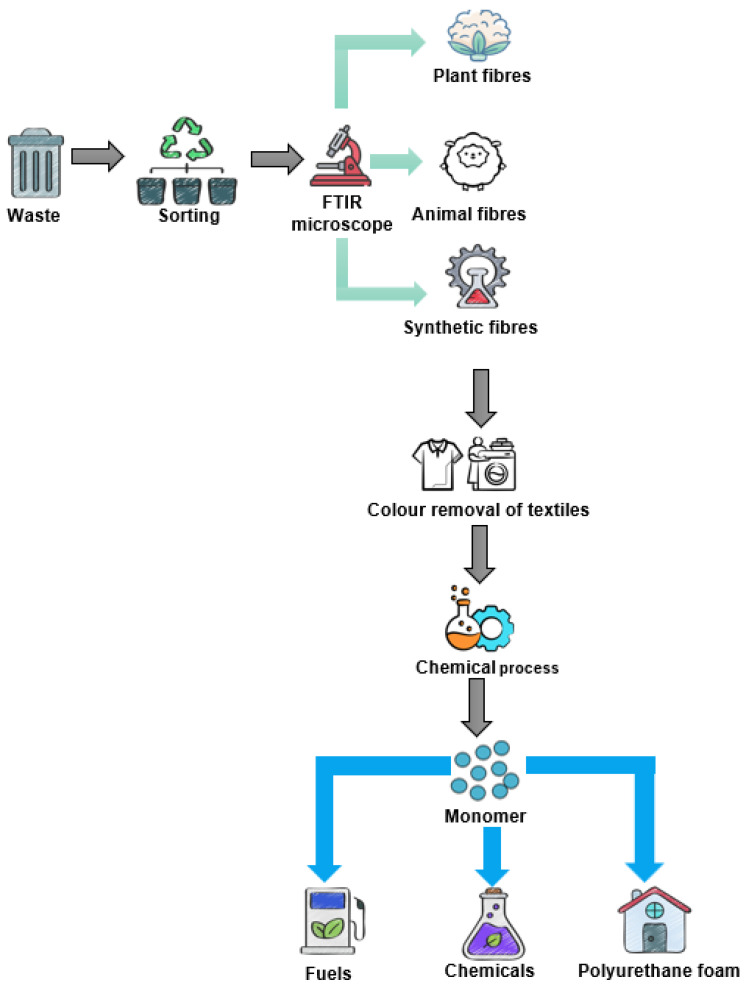
Chemical recycling routes of synthetic textile waste [35].

Textile recycling is typically classified as mechanical, chemical, or biochemical recycling. Mechanical recycling is the processing of textile waste into secondary raw material without significantly changing the polymer chemical structure of the fibres. The steps of mechanical recycling involve collection, identification, sorting, grinding, carding, blending, and re-spinning into new fibres. Mechanical recycling is a waterless process due to the limited need for chemical processing such as dyeing, as the fibres are sorted and recycled with colours from their previous dyes. However, mechanical recycling suffers from the fact that it requires high-purity feedstock, and the inter-fibre friction gradually decreases the quality of recycled fibres, thus reducing their performance during yarn and fabric production [39]. Chemical recycling enables a circular polymer economy by converting waste polymers into virgin monomers, fuels, or other valuable chemicals (Figure 2). Producing monomers in this manner allows for the production of polymer fibres of equal or superior quality compared to virgin materials [40]. However, chemical recycling currently struggles with the presence of blends/multi, as well as chemicals and additives such as dyes and anti-wrinkle agents, which obstruct the process. Biochemical recycling is a process that uses enzymes to depolymerize end-of-life textiles into monomers, which can subsequently be used in the production of value-added products such as glucose and bioethanol [41,42]. The biochemical process requires low energy, uses benign solvents and chemicals, and is based on renewables rather than fossil carbon. In addition, the process is highly selective, making them a good option for separating blended textile waste [43]. Moreover, the biochemical process is costly and slower compared to the chemical recycling process. Even though fibre-to-fibre recycling processes are being developed, these processes are, in reality, downcycling processes in which the mechanical properties of the textile fibres are diminished greatly with each cycle. Therefore, chemically recycling textile fibres that are unfit for other types of recycling or resale due to their poor quality represents a new alternative to completely close the fashion loop. Although there have been extensive research studies on the chemical recycling of different polymers, most of them focus on single-component primary resins or consumer products.

This manuscript will focus on reviewing the most recent results in the chemical recycling process of polyethylene terephthalate (PET), nylon, and elastane textiles; these polymers account for over 80% of the synthetic materials discarded in textile waste [44,45]

### 1.4. Synthetic Polymers

Table 1 illustrates the chemical structure and main applications of the main synthetic fibres used in the fashion and textile industry.

#### 1.4.1. PET

PET is a polymeric material widely used in various applications (textiles, adhesives, coatings, and plastics) that exhibits excellent properties such as light weight, transparency, cost-effectiveness, and high mechanical strength [46]. Consequently, the quantity of PET waste is very significant, and therefore so is its detrimental impact on the ecosystem [47,48]. It is therefore essential to develop sustainable and economical techniques for recycling PET fibre waste for a long-term protection of our resources and the environment. Even though PET represents the largest yield of a polycondensation polymer, most research studies have focused on the chemical recycling of polyester bottles [49,50,51]. Bottle consumption is, in fact, only a small proportion of the total PET production. The large proportion (more than 70%) of PET produced is used to generate fibres and to prepare various textiles by blending with other fibres such as spandex, nylon, and cotton [52,53,54]. The single-component feature of PET bottles has led to an increase in their recycling rate (over 80%) in many countries, but the recycling rate of polyester textiles is less than 20% due to their multi-component feature.

#### 1.4.2. Nylon

The textile industry may be dominated by PET fibre; however, other synthetic polymers also contribute to the fast fashion market and require more attention. Nylon and elastane are the other two synthetic polymer fibres that are used in the manufacturing of polyester textile and that can chemically be recycled. Nylon is a type of polyamide and a thermoplastic polymer which uses the same recycling routes as PET, while elastane is chemically recycled. Nylon is widely used in the fashion and textile industry in the manufacturing of swimwear, activewear, and other performance garments due to its durability and stretchiness. Nylon 6 and nylon 6.6 account for the majority of synthetic polyamides produced worldwide [44,55]. Nylon 6, also known as polyamide 6, is a type of synthetic polymer that is widely used in various applications due to its excellent mechanical properties and versatility. Nylon 6.6 (poly-hexamethylene diamine adipamide) fibre is a member of the large group of polycondensation products of dicarboxylic acids (adipic acid) and diamines (hexamethylenediamine) with fibre-forming properties. Nylon 6.6 fibres are drawn or stretched in a process that increases their length and reorients the fibre’s molecules parallel to one another to generate a strong and elastic filament. The thermoplasticity of nylon enables permanent crimping or texturing of the fibres and provides bulk and stretch properties. In 2022, nylon made up about 5% of the global fibre market [1,5,55]. However, due to technical recycling challenges and comparatively lower prices for virgin fossil-based polyamide, recycled nylon only made up 2% of the total polyamide market share [5,56]. Nylon is the second most used synthetic fibre and represents a significant lever for improved environmental impact if producers switch to recycled and bio-based polyamide. The majority of recycled polyamide originates from either pre-consumer waste, discarded fishing nets, or carpets; however, there is a need to use more of this textile waste as feedstock for the production of other materials. The recycling of polyamides helps to reduce waste, as well as the textile industry’s dependence on virgin fossil-based raw materials.

#### 1.4.3. Elastane

The use of elastane has also gained traction in the textile industry, and it is estimated that 1.2 million tonnes of elastane fibres were produced in 2022, which accounted for around 1% of the global fibre market share [1,5,57]. Furthermore, the share of recycled elastane was estimated to be around 2.8% of global elastane fibre production volumes for that same year [1,57]. Elastane, also known as Spandex or Lycra, is a lightweight synthetic fibre that is commonly used in the manufacturing of clothing such as sportswear and swimwear. Elastane is primarily made up of 85% long-chain polymers called polyurethane [58]. These long-chain polymers consist of alternating units of hard and soft segments. The soft chain segments consisting of either polyethers or polyesters provide elasticity and the hard chain segment consisting of aromatic structures provide strength and long-term stability to the elastane fibre. These characteristics provide elastane with an amazing stretching ability and the ability to come back to shape when released. It is one of the main reasons that elastane is commonly mixed with polyester, polyamide, and natural fibres in the manufacturing of stretched fabrics [59,60]. Elastane is a prerequisite for fashionable or functional apparel which is intended to cling to the body and be comfortable at the same time. Elastane fibres are better compared to rubber in terms of their strength, versatility and light weight. Furthermore, as opposed to rubber yarns, elastane fibres can easily be spun into thin fibres, which renders them highly appropriate for many textile applications.

This review presents a comprehensive analysis of current advancements in chemical recycling technologies for key synthetic fibres such as polyethylene terephthalate, nylon, and elastane. The pre-treatment processes, environmental impact, energy costs, and carbon footprint of various recycling methods are evaluated to highlight their crucial role in enhancing recycling efficacy and developing eco-friendly, economically viable processes. By summarising recent progress and ongoing research, this review provides insights into potential improvements and future directions for achieving circularity in the textile industry.

## 2. Pre-Treatment of Textile Waste

**Figure 3:**
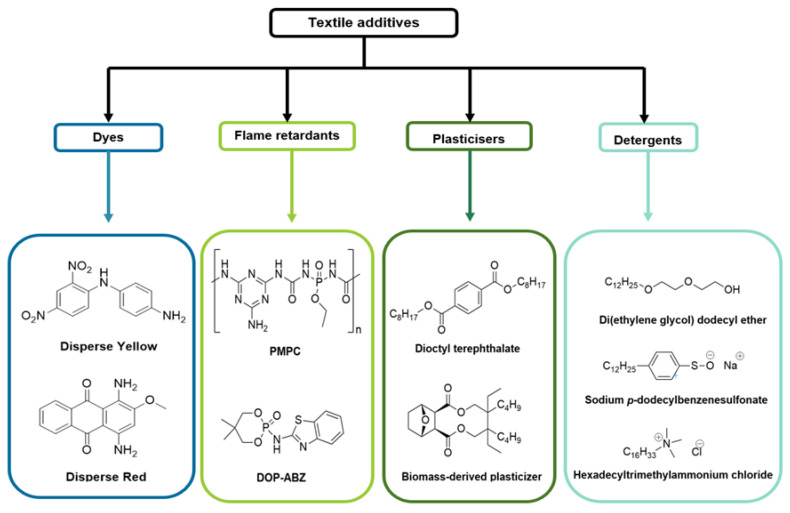
Examples of textile additives including disperse dyes, flame retardants, plasticisers, and detergents.

Pre-treatment is a process applied on textile material to prepare the material for dyeing, printing, finishing or recycling. Pre-treatment plays a crucial role in achieving consistent and high-quality results in textile processing and recycling as impurities can hamper subsequent processes. Thus, making sure that the fabric is clean is crucial to obtain optimum results. The additives present in the natural or synthetic polyester fibres can affect the recycling performance and reduce the quality of recycled products in comparison with bottle grade polyesters [61,62]. These additives provide many features, such as colouring, protection against ultraviolet light, water repellence, and fire resistance. In the textile industry, these additives are typically introduced in the fabric treatment process such as dyeing and finishing, and they are often tenacious as well as resistant to friction and numerous washes [63,64,65]. These particular features render the additives difficult to remove for the recycling process. Synthetic fibres such as polyesters require special dyeing techniques and dyes because they do not contain the reactive functions that enable traditional dyes of natural fibres (cotton and wool) to be used [66,67,68,69]. The developed dyes, as well as flame retardants, plasticisers, and detergents (Figure 3) for polymeric targets, are highly water-consuming and present some health and environmental risks which can interfere with the chemical recycling process if not removed [69,70]. Recently, Shukla et al. demonstrated the successful production of green additives, such as flame retardants derived from bio-macromolecules obtained from lignin for polyester fabrics [71]. Similarly, Sharma and Wazed reported the introduction of bio-sourced functionalities such as antimicrobials on polyester fabrics [72].

### 2.1. Sorting

Synthetic fibre recycling is limited because collection, separation, and sorting are very challenging. Sorting is the critical first step in textile waste recycling. Contrary to the well-organised collection of domestic plastic waste, textiles do not yet have a well-managed dedicated recycling sector. The textile waste collection rates differ from one country to another, while some countries do not have textile-recycling systems [73,74]. The dilemma for textile recycling is sorting and identifying textile materials due to the complex structure of textile polymers. The complicated fabric of the textile renders the sorting and recycling challenging. The fabric waste is commonly mixed with other materials, such as buttons, zippers, or other decorations [75]. Furthermore, the presence of multiple textile components and contaminants increase the difficulty of recycling one targeted material or component. Garments are generally composed of numerous components such as blended fibres, labels, and acrylic which are required to be disassembled manually. There are limited studies reported on sorting of PET textile waste with textile companies favouring the recycling of mono fibres due to cost and tedious processing. Commonly, sorting of textile waste is carried out manually and according to the label specifications. Thus, research aimed at developing sorting and grading systems that quickly sort fabrics based on their fibre type, composition and colours would enable full-scale recycling. From the latest research, the use of infrared and near-infrared spectroscopic methods to detect chemical composition differences between fibre types has shown promise. Automated technologies have a fast response time and can distinguish various textile types even if there are only slight differences among their spectra. Bergfjord and Holst reported that the iso-standardised quantification methods based on distinct dissolution behaviour, such as ISO 1833-1 and morphological variations, found using microscopy can be used to efficiently identify textile materials [76]. Cloud computing technology has become the latest data management solution for sharing manufacturing technology resources and processes involving the textile and plastic bottles lifecycle [77]. These resources can be controlled and managed automatically by applying various internet of things (IoT) technologies, thus making digital sharing easier [78]. An approach for sorting and identifying textile waste has been designed which incorporated the IoT based automatic waste segregator system developed by Lopes and Machado [55,79]. The approach entails detecting and segregating the waste as dry, wet, and metallic waste at the household level and provides real-time monitoring of garbage level in dustbins. The process involves waste materials being sorted through a funnel and a single metallic sensor. The metallic sensor then detects any metal compounds that are going through the funnel. The metal materials go into metal storage and non-metal materials are placed on the sensing platform. The capacitive sensors analyse the object while it is on the sensing platform based on its moisture content. Then, the plastic bottle and textile materials are separated. The sorted textile waste undergoes several sorting and identification processes due to the complex mixture of materials.

Riba et al. developed a fast and accurate multivariate mathematical method for the direct and non-invasive sorting and classification of different textile fibres used for clothing [80]. Zhou et al. also successfully utilised near-infrared to identify different fibre types such as cotton, wool, PET, polylactic acid, and polypropylene. In their study, they used pattern recognition methods to further increase the identification efficiency, and they pre-processed spectra in order to eliminate the baseline shifts which originated from a non-homogeneous distribution of sample quality [81]. Circle Economy and other partner companies have tested out the near-infrared methods using their Fibersort technology [82]. The technique divided textiles into 90 different categories according to their fibre and colour compositions, as well as fabric structure. As a result, they reported a production rate at 1260 kg/h, or one sample per second, which was six times faster than a trained manual sorter. However, this is still slower compared to post-consumer plastics sorting systems.

### 2.2. Cleaning

This is an important step in preparing the fabrics for chemical recycling. Various procedures have been reported which varied in some cases according to the type of fabrics. Using blended PET fabrics (PET/cotton ratios of 45:55, 65:35 and 50:50), Choi and Choi reported the washing of blended fabrics (5 cm × 5 cm) using 1% aqueous sodium carbonate solution. The soaked fabrics were then rinsed with deionised water and dried at 100 °C for 10 min before use [83]. Rosson and co-workers used blended polycotton (PET/cotton ratios of 80:20 and 50:50) and treated it with 0.5 wt% sulfuric acid solution in order to reduce the degree of cotton polymerisation [84]. The solution of sulfuric acid was heated at 75 °C prior to fabric addition and the temperature was kept constant for 1 h and stirring was applied after fabric addition. The pre-treated fabrics (1 cm × 1 cm) were then filtered, rinsed with deionised water, and finally dried in an oven. Peterson et al. as part of their chemical recycling of textile blend from viscose and polyester (70:30 ratio, respectively) either used a standard washing of fabrics then dried it before use or used the fabrics as received [85]. The size of cut pieces of fabrics used in the study was 1 cm × 1 cm. Yousef et al. reported a sustainable green technology for recovery of cotton fibres and polyester from textile waste [86]. In their study, blue jean samples containing 80:20 cotton/polyester and black jean samples containing 100% cotton were used. The samples were washed with soap and water and then rinsed with water to remove any contamination. The washed samples were left to dry at room temperature. The dried samples were then cut into small square pieces (100 mm^2^) for further processing. Zhi reported the pre-treatment of waste polycotton blended fabric for the reconstruction and separation of cotton/polyester blended fabric. The fabric was cut into an area of about 4–25 cm^2^, washed with clean water, and then dried [87,88]. With polycotton (a blend of cotton and polyester) representing one of the most prominent blends on the textile market, Loo and co-workers wrote a review on how to tackle critical challenges in textile circularity by highlighting strategies that could enhance cellulose and polyester recycling from blended fabrics [89]. In their review, they established an in-depth understanding of the technologies needed, with their technical feasibilities as well as their strengths and limitations.

At the time of writing, there are limited available reports on the pre-treatment of mono fibres (100% PET) prior to chemical recycling. Most reports either use standard washing or the fabrics are used as received; thus, more studies still need to be conducted to provide a wide range of washing methodologies. From the available studies, Biswas and co-workers pre-washed the fabrics using a non-ionic surfactant (1% *w*/*w* of Triton-X 100) for 30 min at 50 °C in order to remove any remaining dirt from production [90]. This was followed by a thorough rinsing with distilled water. They then proceeded with an alkaline pre-treatment of the fabrics using a solution of sodium hydroxide (1 M) at 60 °C for 2 h under agitation, followed by extensive washing with distilled water and drying at room temperature. Mehmood et al. also illustrated the pre-treatment of polyester fabric for improved poly-pyrrole adhesion [91]. An undyed PET fabric with 56 ends per inch in warp and 54 picks per inch in weft direction and a PET thin film with 128 mm thickness was used as a reference material. The fabric and thin-film samples were washed in a solution of 5 g/L of ECE Phosphate reference detergent B in water at 50 °C for 30 min. Samples were then rinsed several times with deionised water and then air-dried at room temperature. The cleaned fabric and thin-film samples were conditioned under standard atmospheric pressure at 65 ± 2% relative humidity and 21 ± 1 °C for at least 24 h prior to further process.

### 2.3. Discolouration

The removal of dyes is another important step in preparing the fabric for recycling. It enables the removal of toxins, colourants, flame retardants, or any other contaminants that might affect the chemical recycling process. Dyes and other additives make it more difficult to recycle textile waste than less complex materials such as polyester bottles [92]. The methodology used to recycle polyester bottles cannot be used for polyester clothes because the chemicals and dyes used in the manufacturing of textiles reduce their degree of polymerization in the melting process. Hence, the dye in polyester clothes decreases the quality of the recycled product.

**Figure 4:**
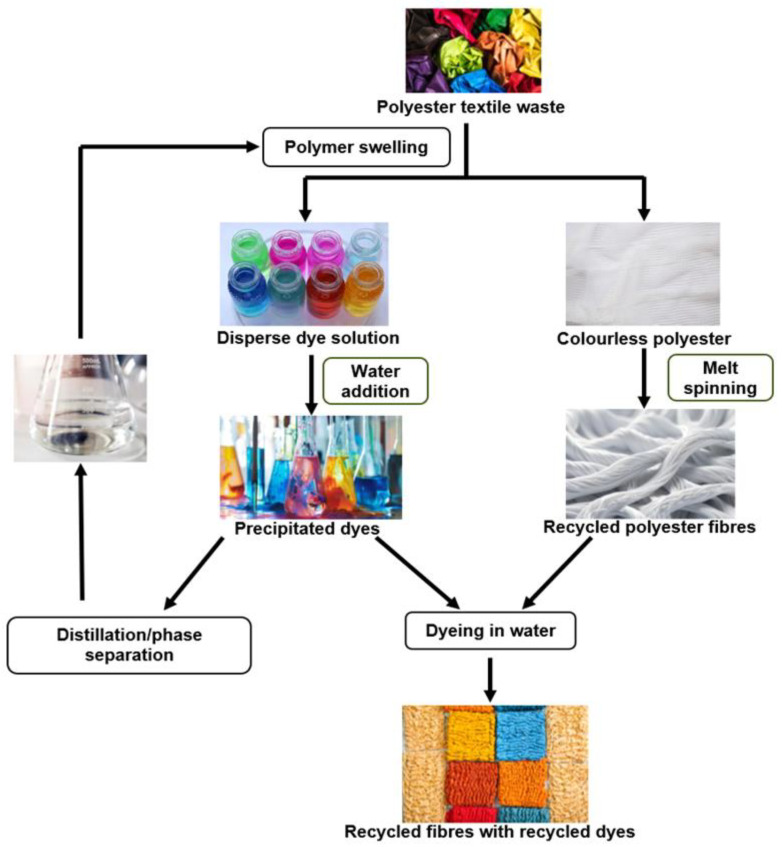
Recycling of polyester and dyes from textile waste [96].

Globally, only 1% of textile wastes, mainly white-coloured wastes, are recycled, and the regenerated fibres’ colour quality is uncontrollable [93,94]. Studies have reported the use of acid, base, or organic solvents for the removal of dyes on fabrics such as cotton, polyester, and polycotton. However, dye degradation methods such as oxidation and photodegradation may damage the polymers and change the dyeability of the regenerated textiles. Yousef et al. demonstrated that 1.0 M of nitric acid (HNO_3_) solution was enough to dissolve and remove the textile dyes from a blue jean sample (80:20 cotton/polyester) after 15 min [86]. At the same time, 1.5 M of HNO_3_ was needed for a black jean sample (100% cotton) after 20 min. Using Yousef’s group findings, Rosson and co-workers pre-treated their blended fabrics (80:20 and 50:50 PET/cotton) with aqueous sulfuric acid and subsequently washed them with deionised water; the dried samples were then treated with a binary solvent system of 80% dimethyl sulfoxide (DMSO) and 20% ionic liquid. The dissolution involved the addition of DMSO under manual stirring before the addition of ionic liquid and subsequent stirring. The solution was then processed in a conditioning mixer (THINKY ARE-250 Mixing and Degassing Machine) for 30 min. After a 24 h period, the solution was filtered using a syringe filter containing a metal mesh with a 15 mm pore size. The cellulose dope filtrate was collected for spinning whilst the solid polyester material was collected, washed, and dried. The recovered polyester was subject to a rinsing step using DMSO to remove residual cellulose solution before being air-dried. Chen et al. illustrated the discolouration of waste polyester textile (100% PET) by hanging a polyester textile sample (5 cm × 5 cm) above the surface of a boiling steam of ethylene glycol [95]. The ethylene glycol steam repeatedly dissolved the dye molecules, while the long chains of the PET molecules were mostly maintained, thus providing an efficient approach for separating small-molecule dyes and long-chain PET molecules. Once there was no change in colour, the textile sample was taken out of the flask and washed with ethanol to remove excess ethylene glycol. Ethanol was then removed using vacuum to afford a discoloured textile. Mu et al. reported the swelling of PET fabric (100% PET) using benzyl alcohol to enable the complete separation of dyes (Figure 4) [96]. The PET fabric was heated at 100–130 °C for a certain period and white PET fibres were obtained. The decolourised PET fabric was then treated with ethanol to remove residual benzyl alcohol and then oven-dried with the benzyl alcohol in solution recycled via distillation. Mu and Yang reported dye removal from PET fabrics (100% PET) using a range of solvent systems such as DMSO, glycerol, ethylene carbonate, tetramethylurea, and a combination of ethylene carbonate and tetramethylurea [97]. Based on the dye removal ratios, they observed that the solvent system of ethylene carbonate and tetramethylurea (7:3 mass ratio) enabled efficient colour separation from polyester fabrics. To completely remove dyes, 2–3 dye removal cycles were required, with each cycle taking place with a liquor ratio of 1:5 at 90–120 °C for 30 min.

The discolouration process can also take place after textile degradation, but this leads to the final colours being unpredictable due to the uncontrollable mixture of colours in the collected textiles. Methodologies have been developed in order to improve the removal of impurities (colours) from the degradation products. Li et al. [98] and Padmanaban et al. [99]. separately reported the use of electrochemical methods during the PET recycling process. In their respective studies, Li et al. [98] used nitric acid-modified activated carbon as an adsorbent for the rapid decolourisation of textile dyes, while Padmanaban et al. [99] illustrated the removal of C.I. Disperse Red 60 from the glycolysis products of waste PET fabrics using ceria and tin-doped zinc oxide nanoparticles. Huang et al. [100] reported an ion-exchange resin that efficiently removes colourants after PET glycolysis. From their reports, the removal rate of the colourant and the retention efficiency of bis- (hydroxyethyl) terephthalate (BHET) was over 99% and 95%, respectively. In addition, they also reported a modified recrystallisation process using ethyl acetate to remove coloured impurities from BHET. The process showed a decolouring rate of over 97.5% for the model-coloured impurities, which was better than the reported physical adsorption processes. Therefore, even though dyes play a significant role in enhancing the aesthetic appeal and marketability of fabrics, there is now a greater push for more sustainable and innovative practices within the textile industry to mitigate their ecological impact.

The pre-treatment of fabrics is a critical step for the efficient and cost-effective recycling of textiles in a non-destructive and environmentally benign manner.

## 3. Post-Treatment of Textile Waste

The pre-treated fabrics can either enter a closed-loop or an open-loop recycling process. A closed-loop process enables the production of new PET materials that could enter the next cycle of textile production. An open-loop process enables the production of various types of materials which can be used in different applications.

In the open-loop recycling process, the second-generation product is commonly not recycled and is disposed of at the end of its lifecycle. This reduces the need to use raw material in the manufacture of second-generation recycled products. Open-loop recycling has proved feasible in the fashion context with regard to pre- and post-consumer textile waste for use in other products, such as bottles used for recycling into textiles. Mechanical recycling has been widely used in the open-loop recycling process compared to chemical recycling. A machine cuts the fabric into smaller pieces and then progressively shreds it until it is in a fibrous state suitable for other processes.

**Scheme 1:**
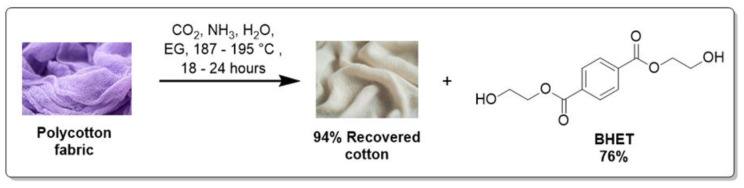
Glycolysis of blended PET with carbon dioxide and ammonia [101].

In the closed-loop recycling process, the recyclable materials are mechanically processed to create a product that performs a similar function. In this process, textile fibres are reused without a loss of properties. Furthermore, the fibres can be recovered from textile waste using numerous mechanical means such as crushing, grinding, and stretching. Then, depending on the fibre origin and the fabric composition, a second series of treatments involving cutting and shredding is applied in order to obtain recycled materials. However, the environmental impact of cutting, shredding, and washing must be taken into account in the life cycle assessment and cost of the new fibres and recycled materials. Another challenge associated with the closed-loop process is the decolourisation of the waste polyester textiles and the purification of the glycolysis product monomer. Yang et al. reported new strategies that enabled a closed-loop process of polyester resources in the textile industry (Scheme 1) [101]. The process comprised the decolourisation of waste textiles, glycolysis of decolourised polyester textiles, purification of crystalline BHET products (76% isolated yield), and polycondensation of the recovered BHET. This new development could be a game changer and facilitate the circular utilisation of polyester resources in the textile industry. Another important challenge of the closed-loop recycling process in the textile industry is the lack of know-how and capabilities to separate mixed-blend fibres. These types of blends (cotton/polyester, viscose/polyester, or cotton/spandex) represent a complex barrier and increase the chemical recycling cost. Loo and co-workers and Choudhury et al. separately catalogued a range of possible techniques which could be used to improve the chemical recycling of blended fibres, thereby enhancing textile circularity within the economy [89,102]. Using a cheap and easily recoverable catalyst is crucial to achieve the closed-loop recycling of PET in a sustainable and cost-effective way, Guo and co-workers reported the efficient depolymerisation of PET using a synthetic nanoclay based on magnesium–aluminium double oxides [103]. The synthetic nanocatalyst is different from conventional catalysts as it is low-cost and environmentally benign. However, they reported that recycling the catalyst was challenging due to its small dimensions, and that the generated monomer retained some catalyst residues, which reduced the quality of the regenerated PET fibre.

Other recycling classification routes include upcycling and downcycling. Upcycling is a term used to describe when the product from recycled materials is of higher value or quality than the original product. Lately, the existing textile recycling routes are in most cases downcycling [104]. For example, clothing and home textiles are downcycled into various products such as low-grade blankets, insulation materials, and industrial rags. Downcycling is a term used to describe when the recycled material is of lower value or quality compared to the original product. Fabric and fibre recycling typically yields materials of lower quality than materials made from virgin fibres. This is due to the length reduction of the fibres and the constituent molecules caused by wear and laundry [105]. Therefore, fabric and fibre recycling is typically considered to be downcycling. In contrast, polymer, oligomer, and monomer recycling typically yields fibres of similar quality to virgin fibres. Considering the current low recycling rate, there is an onus to increase recycling, particularly polymer, oligomer, and monomer recycling, in order to prevent the landfilling or incineration of textile waste that cannot be reused or recycled [106,107]. Even though there have been significant breakthroughs in the separation of polycotton, there remain numerous non-technical barriers to overcome in order to increase textile recycling [107,108]. The recycling of polymers, oligomers, and monomers is dependent on technologies that efficiently sort and separate them into pure fractions.

The post-treatment of textile waste is an important step for the regeneration of new materials that can either be reused for the manufacturing of textiles or used in other processes such as chemical recycling for further applications.

## 4. Chemical Recycling Processes

**Figure 5:**
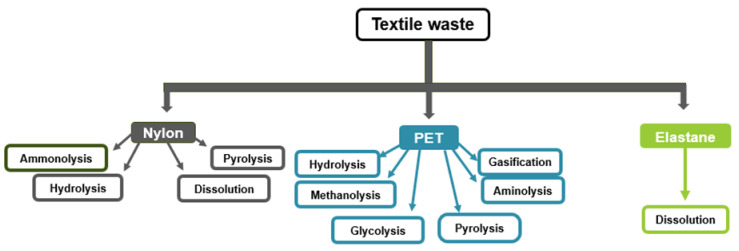
Chemical recycling routes of polymer fibres.

Chemical recycling allows for the degradation of fibres into monomers or building blocks and then re-polymerises them into new fibres without degrading its quality. However, the PET chemical depolymerisation methodology suffers from the use of toxic degrading agents, as well as high pressure and temperatures, which increases its energy consumption [109,110]. In addition, the depolymerisation process generally requires purification and separating steps to afford the product which create issues to the environment. Furthermore, chemical recycling suffers from the presence of blends as well as chemicals and additives such as dyes and anti-wrinkle agents which obstruct the process. Typically, chemical processes are tailored to the polymers found in tissues intended for recycling. For polyesters, the chemical recycling of PET fibre enables the conversion of PET by changing its chemical structure and turning it back into monomers that can be used as feedstocks for the manufacturing of new polymers such as polyurethanes and unsaturated polyester resins. For blended fabrics, monomer recycling is the only recycling path, as contamination prevents polymer recycling through melting and spinning into new textile fibres. Figure 5 illustrates a schematic diagram of the chemical recycling routes of PET, nylon, and elastane.

### 4.1. Polyester Fibre Recycling

Chemical recycling is an attractive process for developing the closed-loop use of polyester materials and reducing the use of virgin polyester materials [111,112]. The most widely reported chemical recycling processes of PET fibre include methanolysis, glycolysis, and hydrolysis. Other reported but less used processes include pyrolysis, gasification, and aminolysis (Figure 5).

#### 4.1.1. Hydrolysis

**Scheme 2:**
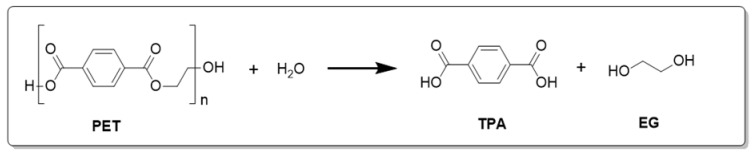
Hydrolysis of PET to generate TPA and EG [113].

Hydrolysis is a chemical breakdown process that occurs due to a reaction with water. Hydrolysis of PET (Scheme 2) affords the degradation of the polymer into terephthalic acid (TPA) and ethylene glycol (EG) [113]. Hydrolysis of waste PET can be carried out under neutral, acid, and alkaline conditions to afford TPA and EG as products [114].

##### Neutral Conditions

In neutral conditions, PET hydrolysis can be achieved with or without a catalyst but often requires high temperatures and reaction times. These reaction conditions represent major disadvantages from the perspective of developing sustainable depolymerisation of PET, particularly in the case of textiles. Sun et al. reported the recovery of high-quality terephthalic acid from waste polyester textiles via a neutral hydrolysis method [115]. In their study, they used a closed-loop route for recycling waste polyester textiles, which comprises pre-discolouration of textile samples, neutral hydrolysis of decolourised waste polyester textiles, and purification of the PTA products under reduced-pressure sublimation. Using this method, the waste polyester textiles were successfully hydrolysed to generate PTA in 95.1% yield and 99.9% purity. The recycled PET produced through re-polymerisation of the recovered PTA exhibited similar properties to those of petroleum-based PET. These results provide a green approach to promoting sustainable development and a circular economy in the textile industry.

##### Acid Conditions

In acidic conditions, PET hydrolysis often requires the use of high temperatures, as well as strong and concentrated mineral acids (sulfuric acid and nitric acid), which favours the development of sustainable processes. Alternative methods using weaker acids or softer acidic conditions still need improvements to achieve the eco-friendly depolymerisation of PET textile wastes. Most studies reporting PET hydrolysis in acid conditions involve plastics, and there are very limited studies on the green depolymerisation of PET textile fibres under acidic conditions. From the available reports, Costa et al. reported the recycling of polycotton textile wastes into new cellulosic raw materials using acid hydrolysis [116]. The selective hydrolysis of cellulose in 1 mol/L aqueous hydrochloric acid or nitric acid under reflux for a few hours led to the recovery of 65–88% of cellulose powder. The cellulose derivatives obtained are very useful as additives in processing industries such as the textile, pharmaceutical, construction, food, plastics, and water treatment industries. Peng et al. reported a new efficient method for acetolysis of waste PET into TPA and EG di-acetate in acetic acid [117]. The main generated components can be reused for further PET synthesis and the life cycle assessment showed that, compared with the existing commercialised chemical recycling methods, acetolysis offers a low-carbon pathway to achieve the full upcycling of waste PET from different sources, including textiles. Compared to neutral hydrolysis, acid hydrolysis is more advantageous due to its low operation costs, and the acid catalyst can be recovered [118,119]. These two acid hydrolysis features have led to recent studies focusing on the design of acid catalysts with more activities than mineral acids for the depolymerisation of PET at higher rates [119].

##### Alkaline Conditions

In alkaline conditions, PET hydrolysis is generally conducted using a metal oxide catalyst such as sodium hydroxide and potassium hydroxide. The base catalyst (potassium hydroxide and sodium hydroxide) is consumed during the process, and the subsequent addition of an acid such as sulfuric acid and hydrochloric acid is performed in order to recover TPA [120]. During the alkaline hydrolysis, the hydroxyl ions from the base attack the electron-deficient carbonyl carbons along the PET main chain, causing scissions at the ester linkages and the production of hydroxyl- and carboxyl-end groups [121,122]. This results in physical and chemical changes in the fibre surface with the formation of small craters and an increasing surface area with extra functional groups. Hydrolysed fabrics have a silk-like appearance and lustre, as well as better wettability and surface area with extra functional groups. However, alkaline hydrolysis has a negative impact on the fabric strength and requires high energy and water consumption [123,124,125]. Čorak et al. reported the alkaline hydrolysis of PET fibres in fabric in a sustainable and energy-efficient way [126]. They demonstrated that it was possible to perform alkaline hydrolysis of PET at low temperature and less time with the addition of the cationic surfactant HDTMAC (80 °C, 10 min), which acted as an accelerator. However, due to the use of sodium hydroxide, the process was not fully environmentally friendly, but since good results were obtained at a lower temperature and time, the process was more economically and energetically acceptable compared to the conventional process and therefore more sustainable. Other commercial surfactants such as the fatty acid amine-amide Lyogen BPN and (3-chloro-2-hydroxypropyl)trimethyl ammonium chloride, which is an epihalohydrin, have also been found to accelerate alkaline hydrolysis [127,128,129]. Recently, the use of ionic liquids has been shown to be successful in the depolymerisation of PET fabrics. Musale et al. have reported the effective weight loss of PET fabric when treated in aqueous and methanolic sodium hydroxide solutions containing the quaternary ammonium compound cetyltrimethylammonium bromide, as well as the ionic liquid 1-butyl-3-methylimidazolium chloride [130]. Furthermore, Cao et al. reported the surface alkali de-weighting and dyeing of polyester fabric by a one-bath and one-step process [131]. In their study, cetylpyridinium chloride and ionic liquids were used as accelerants to perform alkaline de-weighting and dyeing of polyester fabric as a one-step process. They found that ionic liquids and the alkyl imidazolium gemini ionic liquid could be used for alkali de-weighting. However, due to the reaction being irreversible, it was essential to control the alkaline hydrolysis for it to result in de-weighting or complete degradation.

Alkaline hydrolysis of PET is the preferred hydrolysis option, as it can be applied to treat PET waste with higher impurities. In addition, alkaline hydrolysis is more economical than methanolysis due to the cheaper operation costs [132,133]. However, base hydrolysis suffers from the low purity of the generated TPA, which requires further purification steps, hence rendering the process solvent-consuming and impacting the environment. In addition, an acidification step is required in order to separate TPA, and the difficulty to separate and recover the catalyst from the reaction medium significantly limits the implementation of the technique on an industrial scale [132,134].

##### Blended-Fibre Hydrolysis

Typically, blended fabrics consist of hydrolysable natural fibres such as wool and cotton and synthetic fibres such as polyesters and polyamides. Commercially, the chemical recycling of blended textile is not widely applied due to the costly pre-treatment steps in removing contaminants and chemicals from the waste. Furthermore, the complexity attached to the blended material requires the costly separation of the synthetic polymer from the natural fibre. Most of the reported recycling technologies targeting blended textile have only been conducted in small-scale projects, with Palme et al. developing a lab-scale efficient route for the combined recycling of cotton and PET from polycotton [135]. In the process, the hydrolysis of the polycotton using 5–15 wt% NaOH in water and at mild temperatures (70–90 °C) afforded the release of the cotton fibre and the degradation of PET to TPA and EG. Similarly, Yang et al. reported a green approach for the successful separation and regeneration of polycotton-blended textile waste [136]. The approach required the use of a deep eutectic solvent (DES) based on metal salt hydrates (ZnCl_2_/H_3_PO_4_/H_2_O), which led to cellulose dissolution and no reactivity towards the polyester fibres. The environmentally friendly nature of the DES system resulted in the separation of products not requiring complex post-treatment. Furthermore, using 50/50 polycotton t-shirts and zinc oxide as the catalyst, Andini et al. performed time-dependent experiments at various temperatures in order to establish the effect of cotton on polyester glycolysis [137]. Their studies showed that only the polyester reacted, while the cotton remained intact. The cotton did not participate in the glycolysis and higher temperatures (>180 °C) accelerated the complete depolymerisation of the polyester.

Generally, before the recycling of PET fibres from textile waste, pre-treatment processes using acids, alkaline, and ionic liquids are required. In hydrolysis, pre-treatment is important because it can affect the yield, improve the hydrophilicity, and reduce the complexity of the structures of the textile materials by eliminating unwanted contaminants [138]. Gholamzad et al. reported an effective procedure for the conversion of waste polycotton textile to ethanol and subsequent recovery of the polyester part by alkaline pre-treatment [139]. Their findings illustrate that the simultaneous saccharification and fermentation of textile afforded ethanol in 70% yield after the pre-treatment with NaOH/urea at 20 °C, whereas it was only 36% for the untreated textile. Furthermore, the alkaline pre-treatment of the polycotton, followed by hydrolysis, resulted in the recovery of 98% of the polyester without significant changes in its properties. Jeihanipour et al. also reported a novel process for ethanol or biogas production from cellulose in blended-fibre waste textiles [140]. In their study, the cellulosic part of 50/50 polyester/cotton and 40/60 polyester/viscose blended textiles were separated from non-cellulosic fibres using an environmentally friendly solvent called *N*-methylmorpholine-*N*-oxide. The polyesters were purified as fibres after solvent treatments, and up to 95% of the cellulose fibres were regenerated.

#### 4.1.2. Methanolysis

**Scheme 3:**
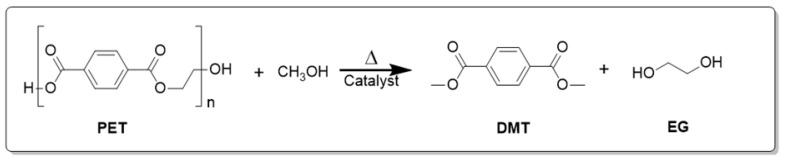
Methanolysis of PET to generate DMT.

Alcoholysis is the depolymerisation of PET in an alcohol medium under high temperature and pressure conditions affording the formation of products with diverse properties [141]. The technique has been developed for over 40 years and is currently the most commercially used methodology for PET recycling [142]. Within the alcoholysis recycling method, methanolysis has gathered more attention in recent years [143,144]. However, the use of other alcohols, such as ethanol, butanol, and trimethylolpropane, has also been reported [144,145]. Methanolysis entails the depolymerisation of PET to dimethyl terephthalate (DMT) using alcohols such as methanol (Scheme 3) as solvents [146,147].

Methanolysis requires mild temperature and pressure conditions. However, the depolymerisation rate is very slow; hence, divalent metal catalysts such as zinc, lead, and manganese acetates are required to enhance this rate [147,148]. Methanol can be used in three separate phases in the PET depolymerisation process: liquid, vapour, or supercritical. Liquid-phase methanolysis is conducted at high temperatures (160–350 °C) and pressures (20–40 atm) in order to afford DMT in high yield [149,150]. The use of vapour and supercritical methanol has also been reported in PET methanolysis, with the latter enabling faster PET depolymerisation in 30 min at 300 °C and at pressures above 80 atm [151,152]. However, these reaction conditions increase the operation costs, hence making the process unfavourable as a direct technique of PET depolymerisation. Both vapour and supercritical methanolysis generate a reaction mixture containing BHET, DMT, and methyl-2-hydroxy ethylene terephthalate, with DMT isolated in 80% yield [153,154]. The separation of DMT from the product mixture contributes to the high costs of PET methanolysis. Zhou et al. [155] and Fang et al. [156] separately reported the use of methanolysis degradation products of waste PET as raw materials for the preparation of waterborne polyurethanes. They demonstrated that the degradation products can improve the thermal resistance, particle size, and stability of viscosity of waterborne polyurethanes. Muangmeesri et al. developed a method for recycling polyesters using an organo-catalysed methanolysis process [157]. The depolymerisation involved the use of benign organo-catalytic (triethylamine) depolymerisation of PET to generate pure DMT and EG. They successfully applied their depolymerisation procedure to other polyesters, polycarbonates, and polycotton. For instance, the successful depolymerisation of polycotton enabled the isolation of cotton, which was further processed into viscose fibres. Furthermore, they also developed a life cycle assessment in order to establish the global warming potential of PET depolymerisation. From their findings, their methodology of PET recycling decreased the quantity of the CO_2_-equivalent emitted by half compared to conventional processes.

#### 4.1.3. Glycolysis

**Scheme 4:**
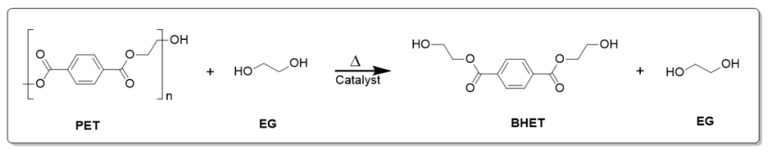
PET glycolysis to generate BHET.

Glycolysis is a degradation process that can be used to convert the large molecules of a textile to small molecules. This process is widely used to recycle textile materials such as PET fibre and polyurethane. Glycolysis involves the depolymerisation of PET to generate BHET with EG or another glycol (Scheme 4) [158,159,160]. The dimer of EG, diethylene glycol, commonly forms in the glycolysis process, which in turn requires separation and purification steps to afford pure BHET [161].

Glycolysis has been industrially used for the recycling of colourless waste bottles [162]. However, despite the high efficiency of glycolysis in recycling polyester waste from single-polymer components, the application of this approach for recovering BHET from waste polyester textiles can be quite challenging due to the heterogeneity of waste polyester textiles. For example, waste textiles are generally composed of blended fibres and contain variable contaminants such as pigments, dyes, dispersants, and various fabrics [163,164,165,166]. Some condensed polymeric fabrics can be converted to the corresponding products during the glycolysis of PET, and these products may further react with the target product BHET to produce second-generation products, which would decrease the purity of the recovered BHET. Therefore, appropriate pre-treatment prior to glycolysis to separate blended fabrics into pure components is essential for the efficient recycling of waste PET textiles. However, this prior separation of the constituent fibres significantly increases the cost of the method. Yang et al. reported a practical and scalable approach for recycling of PET and PET/cotton interwoven fabrics via catalytic glycolysis with ammonium bicarbonate, which decomposes to ammonia, CO_2_, and water [101]. This catalytic approach outperformed conventional acid/base and metal catalysis in selectively recovering and upcycling cotton-based materials. From their studies, they obtained high-purity BHET, which was thermally repolymerised to PET. This method offered a traceless, environmentally friendly, and practical approach for polyester recycling and cotton recovery, representing a significant step toward a sustainable, closed-loop production of plastics and textiles. Similarly, Liu et al. reported the recycling of full components of polyester/cotton blends catalysed by betaine-based eutectic solvents [167]. Polycotton and 100% PET fibre were used in the study and the experiments were performed at a specific temperature and time under atmospheric pressure. PET fibre was fully degraded to afford BHET in 85% yield which could be repolymerised to produce PET, and 95% of the cotton was recycled and maintained its original structural integrity.

Another challenge of the glycolysis technique in recycling waste PET textiles is the discolouration of the waste textiles. Residual dyes stain the recovered monomer, thus reducing its quality and limiting the application of recycled PET fabrics. Therefore, it is crucial to develop a pre-discolouration strategy for waste polyester textiles. Various pre-treatment methods have been developed, such as dissolution methods, which have been used for the separation of PET from blended fabrics by completely dissolving the soluble components [140,168,169]. Therefore, a pre-discolouration process is needed to decolourise the textiles to afford white textiles, which are then subjected to recycling treatment with previously established techniques. This would eliminate or minimise the burden on the subsequent purification of BHET and facilitate the establishment of a closed-loop system of polyester materials in the textile industry. Rosson and co-workers demonstrated an approach to separate coloured cotton and polyester blends, and the dye was retained in the separated polyester and cotton components. The separation was achieved via the chemical dissolution of cotton using a co-solvent system of ionic liquid and dimethyl sulfoxide, from which regenerated cellulose fibres could be directly wet-spun. The polyester component was left intact and recovered via filtration. Furthermore, Mu et al. successfully developed a fibre-to-fibre recycling technology for waste PET textiles [96]. The overall recycling process, which was time-efficient and cost-effective, enabled the recovery of colourless PET in less than 30 min via substantial swelling of PET textiles.

Of the reported chemical recycling processes, glycolysis is the most promising approach for recycling waste PET due to the relatively mild reaction conditions and the weak volatility of ethylene glycol [113,170]. EG is the most widely used degradation agent for PET glycolysis, resulting in the formation of BHET monomers and oligomers. The depolymerisation process involves the glycol diffusing into the polymer, causing the polymer to swell up, thus increasing the diffusion rate. The free lone electron pairs on the oxygen of glycol subsequently react with the carbonyl carbon (C = O) of the PET ester, resulting in the formation of a new C–O bond with glycol while breaking the ester C–O bond. Then, PET is gradually degraded into lower fractions, starting with PET oligomers, then BHET dimers, and lastly BHET monomers [171]. The transformation of BHET dimers into monomers is a reversible process. Thus, prolonging the reaction after the reaction equilibrium has been attained may cause the reaction to shift backwards, thereby increasing the number of dimers at the expense of the BHET monomer [172]. It is therefore important to establish the optimum reaction parameters that allow for high PET conversion, high monomer selectivity, and yield. The PET glycolysis process has a short reaction time and uses minimal energy but is a slow process without the use of a catalyst [173,174]. Recent research has focused on developing high-performance and eco-friendly catalysts that degrade the macromolecules of textile fibres to BHET and other compounds [175,176,177]. In fact, large-scale applications of the microwave-assisted depolymerisation of PET in a continuous manner have shown great potential for recycling, as this method facilitates the depolymerisation of a large amount of material in relatively mild conditions. Zangana and co-workers reported a simple PET glycolytic process that combines an environmentally friendly and cheap heterogeneous catalyst, calcium oxide, with microwave irradiation to obtain the monomer BHET, which can be easily separated by crystallisation [178]. After optimisation, the depolymerisation of post-industrial textile PET waste was achieved in 3.5 min under atmospheric pressure and obtained highly crystalline BHET with a yield of 75%. Luo et al. reported the economic and environmental benefits of modular microwave-assisted depolymerisation of PET [179]. They demonstrated that BHET obtained using microwave irradiation could have lower production costs and emissions than the traditional DMT route due to the high reactivity and excellent reusability of the nano-catalyst zinc oxide. The procedure afforded BHET in 93% yield at 210 °C in 10 min using PET fabric. Egan et al. reported the enzymatic textile fibre separation for sustainable waste processing [180]. In their work, they developed a laboratory scale process for deconstructing and separating cut fabrics into different fibre fractions to create purified product streams that could promote textile recycling. The method involves the combination of aggressive mechanical agitation together with cellulase-catalysed hydrolysis, which led to the complete degradation of the fabric into a slurry and the formation of water-soluble degradation products.

#### 4.1.4. Other Recycling Processes

Other, less used chemical recycling processes have also been reported. However, some of these processes have a limited degree of application.

##### Pyrolysis

**Scheme 5:**
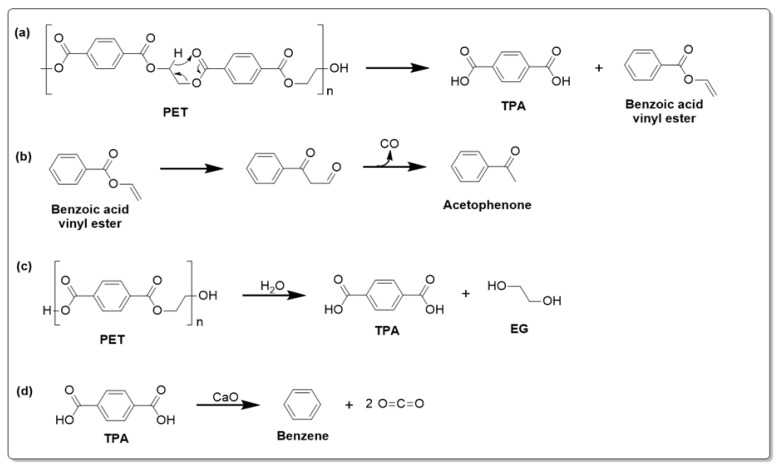
(**a**,**b**) Thermal pyrolysis of PET and benzyl acid vinyl ester; (**c**,**d**) catalytic steam pyrolysis of PET and TPA [181].

Pyrolysis is a thermochemical recycling process of PET that uses high temperatures and pressures. The pyrolysis process, also called dry distillation, is commonly conducted in a tube or autoclave reactor. Small particles of PET and a catalyst are then placed into the reactor vessel set at 370–500 °C. The reaction generates a liquid product which is collected through the cold trap and a gas product that goes through a gas washing bottle, and which is then collected as either waste or gas for the petrochemical industry. This recycling technique is commonly used for plastic recycling, with limited reports on its use in PET fabric recycling. Du et al. reported the conversion of PET-based waste carpet to benzene-rich oils through thermal, catalytic, and catalytic steam pyrolysis (Scheme 5) [181]. Their studies showed that the thermal pyrolysis of waste carpet generated large quantities of benzoic acid and acetylbenzoic acid, whereas catalytic pyrolysis enhanced the decarboxylation of these acids, thereby generating aromatic hydrocarbons. In addition, catalytic steam pyrolysis generated a pure benzene product, which presented a unique opportunity for the reutilisation of this unsustainable waste. Özsin and Pütün studied the co-pyrolysis of lignocellulosic biomass (walnut shell and peach stones) with PET, polystyrene, and polyvinyl chloride [182]. Their comparative studies showed that blending of PET, polystyrene, and polyvinyl chloride into biomass significantly improved the yield of products (benzoic acid and vinyl benzoate).

##### Aminolysis

**Scheme 6:**
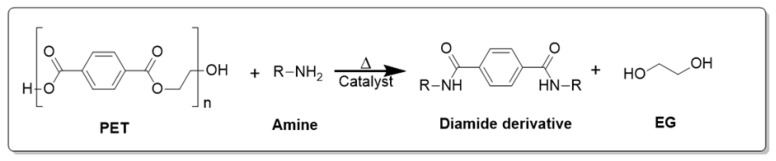
Aminolysis of PET.

Aminolysis is a chemical recycling process of PET that has been little explored as compared to other chemical recycling processes. The aminolytic modification processes of PET fibre surfaces are frequently conducted using primary amines such as ammonia, methylamine, ethylamine, and butylamine to generate terephthalamide derivatives (Scheme 6) [183,184]. Other less commonly used amines include ethanolamine and triethylenetetramine.

The aminolysis process requires milder reaction conditions as opposed to the alcoholysis process, and this is due to the higher nucleophilicity of the amine group over the hydroxyl group [184,185]. Furthermore, the use of homogeneous or heterogeneous catalysts combined with microwave irradiation improves the aminolysis efficiency by reducing the reaction times and temperatures while improving product yields. There are two main approaches in PET aminolysis. The first approach consists of the partial degradation of the polymer, which gives different or even improved mechanico- and physico-chemical properties to the PET fibres. The second approach consists of a complete depolymerisation, which leads to the formation of terephthalamide-based monomers. The latter approach favours upcycling recycling, either by transforming the obtained monomers for uses as building blocks or for polymer reprocessing. To date, only the partial aminolysis process has been developed at the industrial scale, while complete aminolysis is still being conducted at the laboratory scale. Partial aminolysis methods enable surface derivatization while preventing polymer degradation, which then makes it possible to easily place numerous functional moieties onto the surface of the PET-based fabrics [186,187,188,189]. For example, dyeing, antibacterial groups, and hydrophilic functions can be introduced, and these modifications do not significantly affect the mechanical properties of the treated fabrics compared to virgin fibres. In the available literature, the complete depolymerisation approach mostly centres around the catalytic aminolysis process involving PET flakes from bottles [190,191,192]. Some of the most used catalysts in the process involve the acetate, sulphate, carbonate, and bicarbonate salts of metals such as sodium, potassium, zinc, or lead [192,193]. Therefore, aminolysis presents a high potential for PET upcycling and PET fibre functionalization rather than recycling. And even though the technique suffers from the use of large quantities of toxic amines and high temperatures, it also offers many possibilities to generate new monomers for polymer chemistry or other applications. The development of sustainable aminolysis of PET textiles would lead to the process being considered suitable for industrial applications.

##### Gasification

The gasification process recycles textile waste at high temperatures (400–1000 °C) and under either low-oxygen or oxygen-free conditions. Similar to pyrolysis, gasification generates gases with high concentrations of carbons and hydrogens. The gasification and pyrolysis of textile waste under CO_2_ atmosphere are some of the best methods that reduce textile waste and GHG emissions in the atmosphere according to the following equations:

(1) $$Waste\;textile\overset{Pyrolysis}{\to }C_{n}H_{m}+CO+H_{2}\;\Delta H_{1}>0$$

(2) $$C_{n}H_{m}+CO_{2}\overset{Gasification}{\to }CO+H_{2}\;\Delta H_{1}>0$$

However, unlike the pyrolysis process, the gasification process involves chemical reactions with the substrate itself [194]. Synthesis gas, which is commonly known as syngas, is the preferred final product in gasification but ash as well as the by-products of ethylene can also be produced. Syngas is essentially made of hydrogen, CO_2_, carbon monoxide, and methane. The use pressure in the gasification process leads to an increase in yield, calorific value as well as carbon monoxide and hydrogen concentrations while the high temperatures lead to an increase in synthesis gas production and a decrease in char formation.

Gasification has predominantly been used in the textile recycling of polyester, polycotton, and woollen fabrics. Cañete Vela and co-workers reported the potential of feedstock production from the steam gasification of textile waste (cotton, polyester, and polycotton) through experimental work in a fluidized bed bench-scale reactor at high temperature (800–1000 °C) [195]. Their findings showed that both syngas and base chemicals (benzene, toluene, xylene, and styrene) could be recovered from the process. In addition, cotton was more suitable for syngas production, while polyester and polycotton generated both syngas and base chemicals. Wen et al. evaluated the performance of pyrolysis and gasification of waste textile (polyester, cotton, and wool) under CO_2_ atmosphere [196]. The various tests on textile waste were conducted in a thermogravimetric–Fourier transform infrared spectrometer at atmospheric pressure and at 300–500 °C for pyrolysis and 800–1000 °C for gasification. Their findings showed that syngas was generated after treatment with the respective textile waste. Furthermore, a kinetics study showed that the pyrolysis activation energies for the polyester, cotton, and woollen textile waste were 162.38, 103.05, and 110.02 kJ/mol, respectively, while the kinetic parameters of the char particles which were estimated using the shrinking core model showed char gasification activation energies of 178.15, 144.49, and 79.37 kJ/mol for polyester, cotton, and wool, respectively. Using Wen’s group studies, Wu et al. reported the catalytic pyrolysis and gasification of textile waste (polycotton, cotton, and wool) under CO_2_ atmosphere with composite Zn-Fe catalyst [197]. Their findings showed that zinc oxide had the highest catalytic effect on blended textile in the pyrolysis process, while iron(II) oxide had the highest catalytic effect on the char gasification process. In addition, the quantitative evaluation of the pyrolysis and gasification processes using a shrinking core model showed that the pyrolysis activation energies for textiles were 65.1 kJ/mol with 5 wt% ZnO loading, while the gasification activation energies for textile char were 89.0 kJ/mol with 5 wt% iron(II) oxide loading. Therefore, with the use of catalysts lowering the activation energies and the catalytic effect of the composite catalyst being better than that of a single catalyst, these findings could be beneficial to the development of energy utilisation of waste textile under CO_2_ atmosphere.

### 4.2. Nylon Fibre Recycling

Nylon uses the same recycling routes as PET, but it commonly involves polyamide plastic. Nonetheless, there have been reports on the chemical recycling of nylon textiles with economic and environmental benefits. Nylon has similar recycling options as polyesters and can be chemically recycled to its monomer caprolactam. The recycling process of nylon is environmentally benign, but the process is more expensive than the production of virgin caprolactam. Nylon is commonly present in carpets and fishing nets, and these materials are important feedstocks for its recycling [44,55,198]. Recycled nylon exhibits similar features relative to the commercial nylon 6. Furthermore, the recycling of carpets is made possible by blending numerous carpet components through a reactive extrusion and compatibility process which yield lower quality products [198]. Moreover, recycling nylon fibres is more expensive than recycling PET fibres due to the low number of nylon suppliers and recyclers. The lack of an economically viable separation technology has resulted in carpet waste being landfilled and thus creating environmental and health concerns. Therefore, it is urgent to develop a sustainable and cost-effective separation and purification technology to enable carpet recycling on a large scale. The majority of the nylon recycling processes are centred around the nylon plastic fibre and powder. However, some of the reported recycling techniques used in the textile industry are hydrolysis, pyrolysis, dissolution, and ammonolysis.

#### 4.2.1. Hydrolysis

**Scheme 7:**
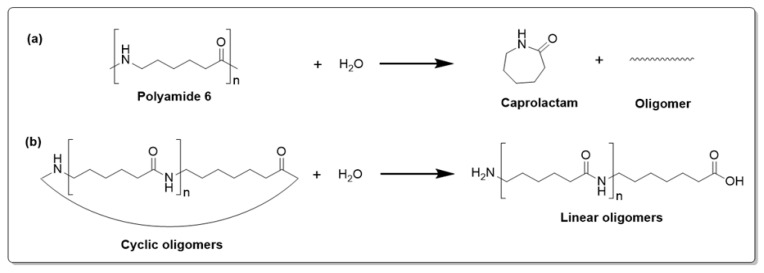
Hydrolysis of (**a**) polyamide 6 and (**b**) waste polyamide 6 textiles into their constituent monomers [199].

The hydrolysis process can be applied to nylon 6 and nylon 6.6 and is conducted at high temperatures and pressures. The process involves the attack on the carboxyl amide by water molecules and is conducted under neutral, acid, or basic conditions. In neutral hydrolysis conditions, Hu et al. reported the rapid hydrolysis of waste and scrap polyamide 6 textiles to generate *ɛ*-caprolactam (Scheme 7) [199]. Their efficient and simple depolymerisation generated high-purity caprolactam (90% yield) in short reaction time (1 h), at high temperature (300 °C) and with low water consumption. Braun et al. reported the depolymerisation of nylon 6 carpet to caprolactam in the presence of steam under medium pressure (800–1500 kPa) [200]. Their observations showed that caprolactam was obtained in 89.7% yield at 340 °C with 6 g/minutes of steam rate and 1500 kPa for 3 h.

In acid conditions, the acid catalyst lowers the activation energy, which enables the breaking of the polymeric chains. The most commonly used acid catalyst is orthophosphoric acid, which is a cheap and strong dehydrating agent. Other less used acid catalysts, such as sulfonic acid and boric acid, are too expensive. The acid hydrolysis process is predominantly applied to nylon 6 and it is currently the only technique applied on a large scale for the depolymerisation of post-industrial and post-consumer nylon 6. The acid-catalysed depolymerisation of nylon 6 generates caprolactam. With regard to the reported acid hydrolysis involving nylon 6.6, Cribbs and Ogale investigated the hydrolytic degradation of nylon 6.6 pile carpet fibres [201]. Their study established the successful degradation of nylon 6.6 carpet fibres under steam-treatment for 5 min and using *p*-toluene sulfonic acid solution. Wang et al. reported the moderate hydrolysis of nylon 6 fabric with different acids [202]. Their investigation illustrated that the content of amino groups, moisture absorption, dyeability, and hydrophilicity of fabrics improved after moderate acid treatment. The acid treatment affected the structural and thermal properties of the nylon fibres and catalysed the hydrolysis of the amide functional groups. In basic conditions, the hydrolysis process involves cleaving the amide bonds and recovering the monomers, caprolactam, and 1,6-hexanediamine. However, the base hydrolysis of nylon has only been reported in the recycling process of nylon plastic fibres, with chemical plants using this technology in order to recover polymer scraps.

#### 4.2.2. Pyrolysis

**Scheme 8:**
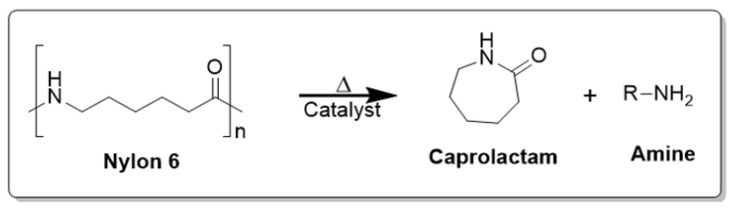
Pyrolysis of nylon 6 to form caprolactam.

The pyrolysis of polyamides has been reported, with the reaction only involving nylon 6 and conducted at temperatures above 300 °C, under an inert atmosphere, and without water (Scheme 8) [203,204]. The reaction mechanism involves an intramolecular cyclisation which leads to the formation of caprolactam. The inert reaction conditions enable the equilibrium to shift towards the formation of the desired product, caprolactam, which can be isolated from the reaction medium in 90% yield. Basic catalysts such as sodium carboxylate and potassium carbonate can be used to speed up the pyrolysis reaction and induce the deprotonation of the amide group, thereby favouring internal cyclisation. Even though pyrolysis appears more attractive than hydrolysis, it is currently not applied at the industrial and commercial level. Czernik et al. conducted a catalytic study of the pyrolysis of nylon 6 from carpet waste material in a bench-scale fluidised bed reactor system for the recovery of caprolactam [205]. The results showed that the reaction proceeded at a high rate and selectivity at 330–360 °C and in the presence of an α-alumina-supported potassium hydroxide catalyst. Furthermore, only a few minutes were needed to depolymerise nylon 6 to caprolactam with 90% purity and 85% yield. Eimontas et al. investigated the catalytic pyrolysis of nylon 6 waste from fishing nets over a zeolite catalyst [206]. Their observations showed that 20 wt% of zeolite catalyst had a significant effect on the yield of volatile products, especially caprolactam. Recovered caprolactam can be used in the manufacturing of other materials such as nylon fibres, carpets, and textiles. In addition, the linear and nonlinear isoconversional can be used to calculate the kinetic parameters and the catalytic pyrolysis of waste from fishing nets. Skvorčinskienė et al. conducted a thermal analysis of fishing net waste and reported that the main polymer of the fishing net was nylon 6, and the material decomposed at a temperature range of 400–440 °C [207]. Furthermore, the analysis of the evolved gas showed that the pyrolysis of nylon 6 resulted in the reduction of the material into monomers and the production of caprolactam. The study offered an innovative waste recycling technique that could help reduce the amount of waste dumped into landfills.

#### 4.2.3. Dissolution

**Figure 6:**
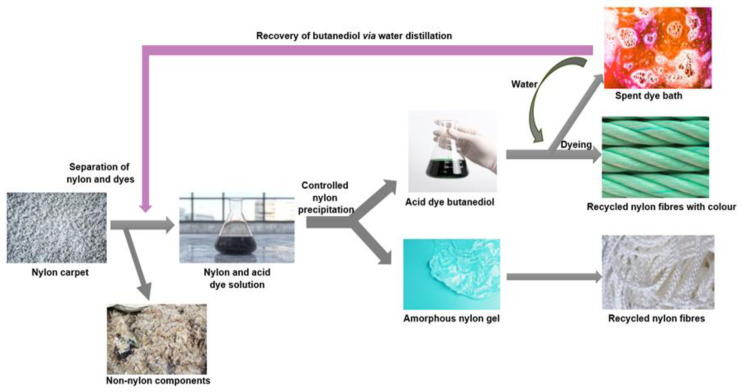
Recycling process of nylon and acid dyes from nylon carpets [209].

The dissolution process involves the selective dissolution of polymeric materials or impurities using a solvent or a mixture of solvents. After filtering the residual nylon and impurities, precipitation can either be induced by using an anti-solvent or lowering the temperature. The polymer obtained by this process is purer than the starting material and is suitable for further recycling. Shukla et al. investigated the depolymerisation of nylon 6 waste fibres by dissolution [208]. Their report showed that heating the waste with formic acid depolymerised nylon 6 into low-molecular-weight compounds of various chain lengths. The study showed that it may be possible to recycle nylon 6-based tyre cord yarn and fishing nets with higher molecular weight chains and reuse the depolymerisation products after conversion to lower-tenacity fibres for fibrefill in pillows or mattresses. Mu et al. investigated the high-quality separation and recovery of nylon and dyes from waste carpet using a dissolution and controlled precipitation approach for sustainable recycling (Figure 6) [209]. From their observations, the recycling process enabled the complete recovery of nylon and dyes in good physical condition. In addition, the overall process was completed in approximately 10 min, with a low energy consumption and minimal waste generation, which rendered the approach potentially profitable and economically viable for nylon recycling.

**Figure 7:**
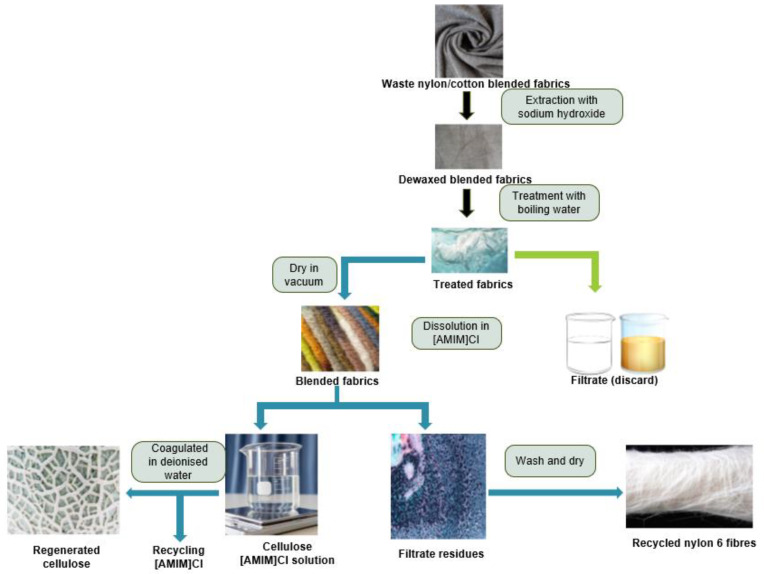
An isolation of cellulose and recycled nylon 6 fibres from waste nylon/cotton blended fabrics [210].

One possible process for fibre recycling is fibre recovery. In this process, the textile is disintegrated into loose fibres, which can be spun into a yarn again. The separation and recycling of waste nylon/cotton blended fabrics as raw materials for different products are attractive. The development of a simple process for separating cellulose and nylon 6 from their blended fabrics is indispensable for the recycling of waste mixed fabrics. Lv et al. successfully isolated and recovered cellulose from waste nylon/cotton blended fabrics using an ionic liquid (Figure 7), 1-allyl-3-methylimidazolium chloride ([AMIM]Cl) [210]. Their findings showed that 58% and 39% of cotton and nylon 6 were recovered, respectively, with 3 wt% waste nylon/cotton fabrics in [AMIM]Cl at 110 °C. Their approach showed that the potential to recycle waste nylon/cotton fabrics into value-added products provided an attractive option for environmental and economic benefits.

#### 4.2.4. Ammonolysis

**Scheme 9:**
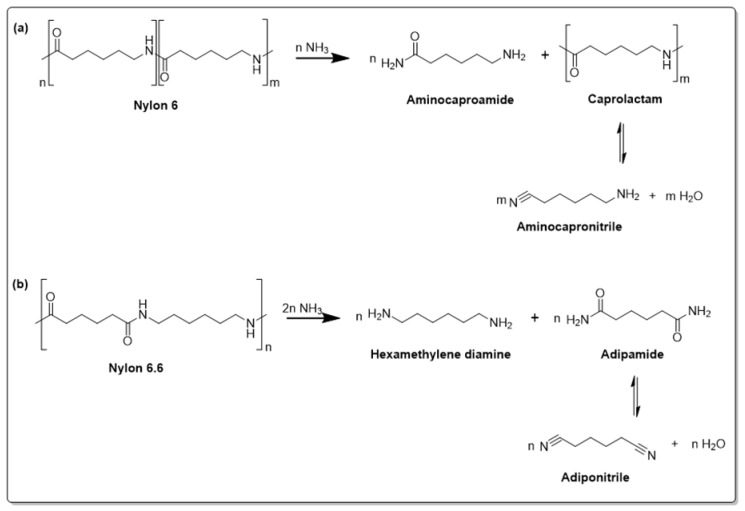
(**a**) Ammonolysis of nylon 6 and (**b**) nylon 6.6 fibres [211].

The ammonolysis process is a process where the nylon polymer chains (nylon 6 and 6.6) are broken due to a transamidation reaction with ammonia at high temperatures (280–330 °C) and pressures. The process yields hexamethylene diamine using nylon 6.6 and caprolactam using nylon 6 as major products, respectively (Scheme 9) [211]. The ammonolysis reaction mechanism of nylon involves the breaking of the amide bonds, followed by dehydration reactions. For nylon 6, the dehydration step involves ring addition and ring opening reactions for the conversion of the cyclic lactams. The process can generate products with over 90% yield if the non-converted oligomers are recirculated. Ammonolysis is the primary depolymerisation method of nylon 6.6 carpet waste. With most reports on ammonolysis recycling of nylon involving plastics and powder, Duch and Allgeier reported a nylon recycling process of carpet waste via ammonolysis that deactivated a Raney catalyst [212]. Their findings showed that nylon 6 and nylon 6.6 fibres and polymers could be recycled via a combination of ammonolysis followed by dehydration and nitrile hydrogenation to generate hexamethylenediamine. During the process, the Raney–Cobalt catalyst was not deactivated, while the Raney–Nickel catalyst was deactivated due to the formation of oligomers arising from the amine condensation reactions. Catalyst deactivation could be reversed with the addition of either ammonia or sodium hydroxide.

### 4.3. Elastane Fibre Recycling

**Figure 8:**
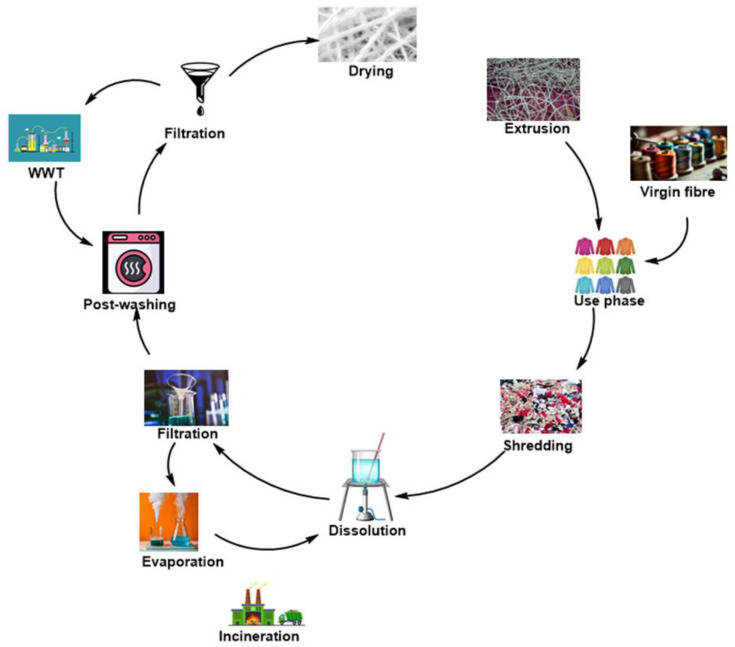
Life cycle assessment of elastane dissolution from textile blends [214].

Elastane is usually present in fabrics in only a very small proportion, making its recycling challenging and causing it to interfere in the recycling of other polymer types in clothing. The complex molecular structure of elastane also renders its recycling challenging, with the available literature highlighting dissolution as the only viable process to separate it from blends. In fact, elastane is usually used in blended fabrics and can be removed from the fabric in order to facilitate the recycling of other fibres. As the use of fabrics containing elastane for apparel applications continues to grow, the need to develop eco-friendly technologies to recycle the industrial and post-consumer waste for elastane blended fabrics becomes important. The typical solvents for elastane dissolution are dimethylformamide and dimethylacetamide, which are toxic and not environmentally friendly, making them difficult to handle in large quantities. Recently, better processes have been developed, with Vonbrül et al. reporting the selective separation of elastane from clothing by dissolution method [213]. Their studies showed that elastane can be separated at room temperature by mixing tetrahydrofuran and dimethyl sulfoxide in 70:30 volume ratio and an ether solvent at a specific ratio. This solvent mixture exhibited low viscosity as well as excellent solubility properties for elastane. Phan et al. analysed the potential of the selective dissolution of elastane from mixed-fibre textile waste [214]. They developed a thermogravimetric analyser-corrected method as a quantification tool and established that tetrahydrofurfuryl alcohol and ɣ-valerolactone successfully dissolved elastane in polyester/elastane and polyamide/elastane blends. In addition, the life cycle analysis study showed that this selective solvent-based dissolution process saved 60% CO_2_-eq./kg textile waste compared to incineration. This interdisciplinary work could enhance further development and upscaling of dissolution recycling processes for blended textiles. In addition, in their studies, Phan et al. [214] also reported the effective selective chemical dissolution of elastane from blended textiles (PET, cotton, nylon, wool, and polyamide). Their study showed that the elastane part of polyester/elastane blends could be removed by selective dissolution in either cyrene, dimethylacetamide, dimethylformamide, or tetrahydrofurfuryl alcohol (Figure 8). The dissolution process involves processing steps such as shredding, washing, drying, and extrusion. Furthermore, the life cycle assessment study showed that the selective solvent-based dissolution is a more environmentally friendly recycling pathway, with 60.3% CO_2_-eq./kg textile waste saved compared to the incineration of textile waste.

Yin et al. reported the eco-friendly removal of spandex from nylon/spandex blended fabrics by selective polymer degradation [215]. Their findings showed that by treating the blended fabric at 220 °C for 2 h under atmospheric pressure enabled the effective degradation of the spandex fibre and the recovery of the nylon component for recycling. The spandex residue was washed off with ethanol and the nylon fibre retained its original morphology. Wang et al. reported the possibility to directly recycle waste cotton/elastane (95:5) blended fabrics through dissolution and regeneration, with the resultant films having potential applications as packaging materials [216]. The blended fabric was successfully converted into regenerated films using aqueous sulfuric acid, aqueous NaOH/urea, and LiCl/DMAc solvent systems respectively. All three solvent systems could effectively dissolve the cellulose fibres, while generating different forms of elastane components. In sulfuric acid solvent, the elastane fibres were partially hydrolysed and reduced the transparency of regenerated films, while in NaOH/urea solvent, they were well retained and interrupted the structure of regenerated cellulose films. In LiCl/DMAc solvent, the elastane fibres were completely dissolved and formed a composite structure with cellulose, leading to improved tensile strength and water barrier property. Villar et al. demonstrated that high quality cellulosic fibres could be engineered from cotton/elastane (95:5) textile waste using aminolysis [217]. The aminolysis of elastane was conducted under mild conditions using dimethyl sulfoxide in combination with diethylenetriamine as the cleaving agent and 1,5-diazabicyclo [4.3.0]non-5-ene as the catalyst. The analysis of the nitrogen content in the recovered cellulose fraction showed that 2 h of reaction at 80 °C was enough to reduce the elastane content to less than 0.08%. In addition, the degraded elastane products were recovered through precipitation with water and the characterisation of the recovered cellulose showed that its macromolecular properties and crystal structure were maintained. Dry-jet wet spinning was used in order to turn the cellulosic component into new fibres with excellent tensile properties. Johansen et al. reported the selective chemical disassembly of elastane fibres and polyurethane coatings in blended textiles [218]. Their findings showed the successful depolymerisation of elastane recovered from the dissolution of fabric blends, as well as the direct disassembly of polyurethane-coated textiles with polyamide fabrics. A solvolysis process with *tert*-amyl alcohol was conducted as part of the dissolution process. This solvolysis process represented an efficient option for the two-step process involving dissolution for fibre separation followed by chemical depolymerisation of elastane. This work provides possibilities to recycle certain blended textiles and apply solvolysis for the removal and depolymerisation of elastane from fabrics for potential repolymerisation and use in new elastane fibres.

The textile industry is on its early steps towards a circular economy. The global rise in population, industrial growth, and improved living standards have led to greater demand for fibre consumption and textile waste generation. Thus, it is essential for the textile industry to embrace fibre-to-fibre recycling in addressing the increasing textile waste problem and incorporate economic and environmental sustainability guidelines for long-term textile waste management. Chemical recycling could provide a sustainable avenue to reducing textile waste with valuable feedstock generated in the process.

## 5. Current and Future Directions

Currently, the textile industry is continuously evolving by catering to fashion, style, and marketing needs. This constant production of fibres and their subsequent disposal operations have generated an environmental hazard to communities and freshwater systems, especially with the release of harmful chemicals during the fibre manufacturing process. With less than 1% of textiles being recycled globally and the majority being either landfilled or incinerated, it is therefore urgent to encourage consumers to make behavioural changes towards their textile waste and develop eco-friendly textile products, as well as recycling techniques for zero-waste production.

The textile recycling process is complex and involves numerous stages such as collection, sorting, processing, and manufacturing. Each stage of the recycling process requires different equipment and expertise which render developing a streamlined and efficient recycling process difficult. A notable obstacle in textile recycling procedures is the absence of dedicated textile collection services in many countries. This issue is particularly troublesome due to the diverse array of materials, fibres, and blends found in textiles, which require specialised recycling processes. Consequently, the accumulation, sorting, and storage of these textiles are critical and the absence of infrastructure for appropriate collection of textile waste significantly impacts the integrity of textile recycling. Innovations that allow for the mechanisation and automation of textile sorting, fibre identification, and the creation of new fibres from pre- and post-consumer textile waste are being developed and implemented as the textile industry moves to address this change. There are also economic and policy barriers that impact the effective implementation of textile recycling. On the economic front, the main barrier is the high cost of recycling textiles. Recycled textile materials are more expensive than virgin materials which makes it costly for manufacturers to use them. Furthermore, the market for recycled textiles is small and fragmented which makes it difficult for producers to find buyers for their recycled materials. Economic incentives should be introduced in order for textile companies to encourage textile reuse and recycling. Similarly, the marketing of sustainable blended materials made from recycled fibres would alleviate the environmental impact of textile waste. On the policy front, the lack of standardisation and clarity around regulations that govern textile recycling led to compliance challenges and high costs. Furthermore, the lack of financial incentives or government subsidies for the adoption of textile recycling technology contribute to the overall economic burden and lower the attractiveness of recycled materials to consumers. Thus, these economic and policy barriers need to be addressed in order to unlock the full potential of textile recycling as a sustainable solution for our environment.

Moreover, efforts have already been made in order to increase awareness of the environmental impact of the global textile complex through scientific reporting, social awareness, and accessibility to information. These efforts are major drivers of moving industry and consumers to a circular economy. Many people are unaware of the environmental impact of textile waste and the benefits of recycling. As a result, they may not prioritise textile recycling and dispose of textiles in their general waste. Typically, consumers’ behaviour toward fashion industry products is different from their behaviour towards other products such as household appliances. It is more common to change household appliances when they break; however, people change clothes for trivial reasons, such as going out of fashion. Other major drivers of recycling are the clothing manufacturers, textile organisations, and governments, which have already made efforts in order to intensify recycling initiatives and policies in the textile industry. In recent years, companies have implemented initiatives to reduce textile waste such as encouraging the use of recycled materials and implementing more sustainable practices in the production process. These changes can help to protect the environment and create a more sustainable fashion industry. Both start-ups and well-established companies have taken leadership roles through all sectors of the textile and apparel supply chain to meet the new sustainable practices in the textile industry. For example, Levi’s have produced party dresses using recycled polyester and organic cotton [12,15,219]. Non-profit organisations such as Textile Exchange have increased sustainability in the textile value chain around the world. In 2012, Textile Exchange introduced the concept of the Global Recycled Standard (GRS), which covers the recycling of pre-consumer and post-consumer waste (not including pre-industrial waste) [57,220]. The GRS is an international, voluntary and full product standard that sets requirements for third-party certification of recycled content, chain of custody, social and environmental practices, and chemical restrictions. The GRS uses the ISO 14021 definition of recycled content and is intended for use with any product that contains at least 20% Recycled Material [221,222]. GRS offers a three-level system based on the total recycled material content in the final product [57,222]. Lindex offers clothing made from either organic or recycled materials and their labelled recycled garments are made from different recycled materials such as polyester, polyamide, or cotton [223]. Governments have introduced legislation related to textile waste, both pre- and post-consumer waste, and in some cases the implementation of Extended Producer Responsibilities. For example, the EU’s Waste Directive Framework requires countries to separate all textile waste by 2025, and the Circular Economy Action Plan ensures that circular economy principles are applied to all textile manufacturing products [224]. Government intervention is crucial and may lead to new value-added recycling technologies entering the market, and therefore changing the current average system. Improving recycling technologies could lead to recycling being even more worthwhile than reuse, especially if the systems become closed-loop.

Along with sustainability awareness and changing consumers’ behaviour efforts, there is a need for research and technological innovation to overcome existing limitations in recycling of textile waste. Research, innovation, and scale-up are needed in developing sustainable technologies for waste sorting in preparation for recycling and for handling blended fabrics, while the existing recycling technologies need to become cleaner, more energy-efficient, and less costly. In the case of textiles, chemical recycling has the potential to provide one of the most environmentally benign options, using methods where limited hazardous catalysts and process conditions are employed. In addition, chemical recycling allows for the return of textile waste to the initial monomers, which can in turn be repolymerised or further modified for use as building blocks for other materials. However, even though chemical recycling is regarded as the key approach to recycle textiles without downcycling the polymers, the process still faces challenges in becoming a competitive route at industrial level. The process suffers from issues with scale-up, cost, organic solvent handling, and the recoverability/reusability of the catalysts. All clothing companies should conduct a life cycle assessment (LCA), which is critical in the textile supply chain, of their recycling process to assess their sustainability. In addition, there is a need to integrate modern technologies such as automation and mobile applications in the sorting and recycling of textile waste. Research can also contribute towards the valorisation of textile waste to value-added products by designing and producing sustainable fabrics, as well as establishing strategies aimed at decreasing waste and introducing new materials and fibres to the textile industry. Thus, sustainable business policies and models are required in order to redesign the next generation of fashion that would enable a smooth transition to a textile system that provides better economic, societal, and environmental benefits. It is absolutely vital that there is an increased collaboration between non-governmental organisations, industry, and academia to drive transformative change in order to tackle the complex issues facing the textile industry.

## 6. Conclusions

Numerous methods and techniques have been reported for the recycling of textiles. Mechanical recycling has been largely demonstrated on an industrial scale but suffers from the lack of recyclability, renewability, and sustainability. Biological and especially chemical recycling provides a perfect alternative for a long-term sustainable circular economy. Chemical recycling enables open- and closed-loop recycling, the use of green chemicals and catalysts, and sustainable procedures. However, textile chemical recycling suffers from issues related to method scale-up and cost, high solvent consumption, and the low reusability of the catalysts. Nonetheless, chemical recycling is currently regarded as the key approach to recycle textiles without downcycling the polymer. Solvolysis methods (glycolysis, methanolysis, and hydrolysis) enable the depolymerisation of textiles into compounds that can be used for either repolymerisation to polymers/oligomers or the synthesis of other products.

## Figures and Tables

**Table 1:** Characteristics and main applications of PET, nylon, and elastane fibres.

Polymer 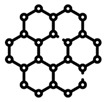	Molecular Structure 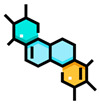	Main Applications 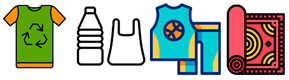
Polyethylene terephthalate	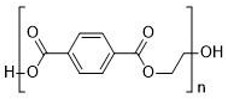	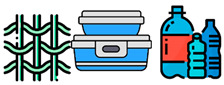 Textile fibres, food containers and plastic bottles
Nylon 6	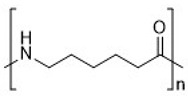	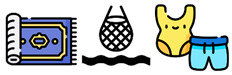 Carpets, fishing nets and swimwear
Nylon 6.6	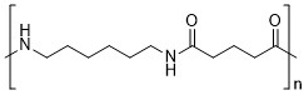	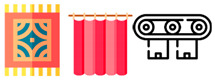 Carpets, curtains and conveyor belts
Elastane	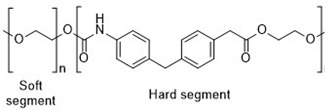	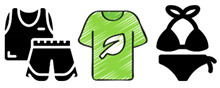 Sportswear, swimwear and textile blends

## Data Availability

Not applicable.

## References

[1] Leal Filho W., Perry P., Heim H., Dinis M.A.P., Moda H., Ebhuoma E., Paço A. (2022). An overview of the contribution of the textiles sector to climate change. Front. Environ. Sci..

[2] Panhwar A., Sattar Jatoi A., Ali Mazari S., Kandhro A., Rashid U., Qaisar S. (2024). Water resources contamination and health hazards by textile industry effluent and glance at treatment techniques: A review. Waste Manag. Bull..

[3] Textile Exchange (2023). Releases Preferred Fiber and Materials Market Report. Outdoor Ind. Compass.

[4] Henry B., Laitala K., Klepp I.G. (2019). Microfibres from apparel and home textiles: Prospects for including microplastics in environmental sustainability assessment. Sci. Total Environ..

[5] Ramasamy R., Subramanian R.B. (2021). Synthetic textile and microfiber pollution: A review on mitigation strategies. Environ. Sci. Pollut. Res..

[6] de Oliveira C.R.S., da Silva Júnior A.H., Mulinari J., Ferreira A.J.S., da Silva A. (2023). Fibrous microplastics released from textiles: Occurrence, fate, and remediation strategies. J. Contam. Hydrol..

[7] Bailey K., Basu A., Sharma S. (2022). The Environmental Impacts of Fast Fashion on Water Quality: A Systematic Review. Water.

[8] Niinimäki K., Peters G., Dahlbo H., Perry P., Rissanen T., Gwilt A. (2020). The environmental price of fast fashion. Nat. Reviews. Earth Environ..

[9] DeVoy J.E., Congiusta E., Lundberg D.J., Findeisen S., Bhattacharya S. (2021). Post-Consumer textile waste and disposal: Differences by socioeconomic, demographic, and retail factors. Waste Manag..

[10] Textile Exchange (2022). Releases Preferred Fiber and Materials Market Report. Outdoor Ind. Compass.

[11] Stanes E., Gibson C. (2017). Materials that linger: An embodied geography of polyester clothes. Geoforum.

[12] Nkatha K. How Fast Fashion Is Fuelling the Fashion Waste Crisis in Africa–Greenpeace Africa. https://www.greenpeace.org/international/story/62308/how-fast-fashion-fuels-climate-change-plastic-pollution-and-violence/.

[13] Micheni K. Fighting the Effects of Fast Fashion in Kenya. https://www.rte.ie/news/2023/0525/1385422-fast-fashion-nairobi/.

[14] Bartlett J. Chile’s Atacama Desert Has Become a Fast Fashion Dumping Ground. https://www.nationalgeographic.com/.

[15] Hyde P. Rags, Not Riches: Why Ghana Is Fast Fashion’s Dumping Ground–Forbes Africa. https://www.forbesafrica.com/fashion/2023/01/18/rags-not-riches-why-ghana-is-fast-fashions-dumping-ground/.

[16] Maxine Bedat See the Horrifying Place Where Your Old Clothes Go to Die–Fast Company. https://www.fastcompany.com/90640931/see-the-horrifying-place-where-your-old-clothes-go-to-die.

[17] Šajn N., European Parliamentary Research Service Environmental Impact of Textile and Clothes Industry. https://www.europarl.europa.eu/RegData/etudes/BRIE/2019/633143/EPRS_BRI(2019)633143_EN.pdf.

[18] Muthu S.S. (2022). Sustainable Approaches in Textiles and Fashion: Circular Economy and Microplastic Pollution.

[19] Balasaraswathi S.R., Rathinamoorthy R. (2022). Synthetic Textile and Microplastic Pollution: An Analysis on Environmental and Health Impact. Sustainable Approaches in Textiles and Fashion.

[20] European Environment Agency Textiles-in-Europe-s-Circular-Economy.pdf. https://www.eea.europa.eu/downloads/e27564dbf0f7462ea5c52e4c9aaf2775/1676462612/textiles-in-europe-s-circular-economy.pdf.

[21] Leslie H.A., van Velzen M.J.M., Brandsma S.H., Vethaak A.D., Garcia-Vallejo J.J., Lamoree M.H. (2022). Discovery and quantification of plastic particle pollution in human blood. Environ. Int..

[22] Liu S., Liu X., Guo J., Yang R., Wang H., Sun Y., Chen B., Dong R. (2023). The Association Between Microplastics and Microbiota in Placentas and Meconium: The First Evidence in Humans. Environ. Sci. Technol..

[23] Ragusa A., Svelato A., Santacroce C., Catalano P., Notarstefano V., Carnevali O., Papa F., Rongioletti M.C.A., Baiocco F., Draghi S. (2021). Plasticenta: First evidence of microplastics in human placenta. Environ. Int..

[24] European Parliament, How the EU Wants to Achieve a Circular Economy by 2050|Topics|European Parliament. https://www.eumonitor.eu/9353000/1/j9vvik7m1c3gyxp/vlgjfy6bvmtu?ctx=vhsjgh0wpcp9.

[25] European Environment Agency, Textiles-and-the-Environment-the.pdf. https://www.cscp.org/wp-content/uploads/2022/03/ETC_Design-of-Textiles.pdf.

[26] Radhakrishnan S. (2022). Sustainable Development Goal: Sustainable Management and Use of Natural Resources in Textile and Apparel Industry. Sustainable Approaches in Textiles and Fashion.

[27] Jeya G., Sunitha T.G., Sivasankar V., Sivamurugan V. (2022). Advancements in Recycling of Polyethylene Terephthalate Wastes: A Sustainable Solution to Achieve a Circular Economy. Sustainable Approaches in Textiles and Fashion.

[28] Pla-Tolós J., Serra-Mora P., Hakobyan L., Molins-Legua C., Moliner-Martinez Y., Campins-Falcó P. (2016). A sustainable on-line CapLC method for quantifying antifouling agents like irgarol-1051 and diuron in water samples: Estimation of the carbon footprint. Sci. Total Environ..

[29] Aliprandi F., Stoppato A., Mirandola A. (2016). Estimating CO_2_ emissions reduction from renewable energy use in Italy. Renew. Energy.

[30] Herva M., Franco A., Ferreiro S., Álvarez A., Roca E. (2008). An approach for the application of the Ecological Footprint as environmental indicator in the textile sector. J. Hazard. Mater..

[31] European Parliament, Emissions from planes and ships: Facts and figures (infographic)|Topics|European Parliament. https://www.europarl.europa.eu/topics/en/article/20191129STO67756/emissions-from-planes-and-ships-facts-and-figures-infographic.

[32] European Parliament, The Impact of Textile Production and Waste on the Environment (Infographics)|Topics|European Parliament. https://www.europarl.europa.eu/topics/en/article/20201208STO93327/the-impact-of-textile-production-and-waste-on-the-environment-infographics.

[33] Li Z., Zhou Y., Zhao M., Guan D., Yang Z. (2024). The carbon footprint of fast fashion consumption and mitigation strategies-a case study of jeans. Sci. Total Environ..

[34] Leal Filho W., Dinis M.A.P., Liakh O., Paço A., Dennis K., Shollo F., Sidsaph H. (2024). Reducing the carbon footprint of the textile sector: An overview of impacts and solutions. Text. Res. J..

[35] Tournier V., Duquesne S., Guillamot F., Cramail H., Taton D., Marty A., André I. (2023). Enzymes’ Power for Plastics Degradation. Chem. Rev..

[36] Sinha P., Dissanayke D.G.K., Abeysooriya R.P., Bulathgama B.H.N. (2022). Addressing post-consumer textile waste in developing economies. J. Text. Inst..

[37] (2020). U.S. Recycling Economic Information Study–Recycling Economic Information (REI) Report; 2020 Recycling Economic Information Report. https://www.epa.gov/.

[38] Khan D., Kumar A., Samadder S.R. (2016). Impact of socioeconomic status on municipal solid waste generation rate. Waste Manag..

[39] Lindström K., Sjöblom T., Persson A., Kadi N. (2020). Improving Mechanical Textile Recycling by Lubricant Pre-Treatment to Mitigate Length Loss of Fibres. Sustainability.

[40] Sandin G., Peters G.M. (2018). Environmental impact of textile reuse and recycling—A review. J. Clean. Prod..

[41] Maheswari N.U. (2016). Biological Degradation of Textile Dyes using Marine Bacillus species. Int. J. Pure Appl. Biosci..

[42] Hu Y., Du C., Leu S., Jing H., Li X., Lin C.S.K. (2018). Valorisation of textile waste by fungal solid-state fermentation: An example of circular waste-based biorefinery. Resour. Conserv. Recycl..

[43] Piribauer B., Bartl A. (2019). Textile recycling processes, state of the art and current developments: A mini review. Waste Manag. Res..

[44] Bianchi S., Bartoli F., Bruni C., Fernandez-Avila C., Rodriguez-Turienzo L., Mellado-Carretero J., Spinelli D., Coltelli M. (2023). Opportunities and Limitations in Recycling Fossil Polymers from Textiles. Macromol.

[45] Stefan D.S., Bosomoiu M., Stefan M. (2022). Methods for Natural and Synthetic Polymers Recovery from Textile Waste. Polymers.

[46] Sinha V., Patel M.R., Patel J.V. (2010). PET Waste Management by Chemical Recycling: A Review. J. Polym. Environ..

[47] Palacios-Mateo C., van der Meer Y., Seide G. (2021). Analysis of the polyester clothing value chain to identify key intervention points for sustainability. Environ. Sci. Eur..

[48] Dadash Ziyaei M., Barikani M., Honarkar H. (2020). Recycling of Polyethylene Terephthalate (PET) *via* Glycolysis Method for Synthesis Waterborne Polyurethane. Eco-friendly and Smart Polymer Systems.

[49] Tournier V., Topham C.M., Gilles A., David B., Folgoas C., Moya-Leclair E., Kamionka E., Desrousseaux M., Texier H., Gavalda S. (2020). An engineered PET depolymerase to break down and recycle plastic bottles. Nature.

[50] Ghosh T., Avery G., Bhatt A., Uekert T., Walzberg J., Carpenter A. (2022). Towards a Circular Economy for PET Bottle Resin Using a System Dynamics Inspired Material Flow Model. J. Clean. Prod..

[51] Babaei M., Jalilian M., Shahbaz K. (2024). Chemical recycling of Polyethylene terephthalate: A mini-review. J. Environ. Chem. Eng..

[52] Raheem A.B., Noor Z.Z., Hassan A., Abd Hamid M.K., Samsudin S.A., Sabeen A.H. (2019). Current developments in chemical recycling of post-consumer polyethylene terephthalate wastes for new materials production: A review. J. Clean. Prod..

[53] Stone C., Windsor F.M., Munday M., Durance I. (2020). Natural or synthetic—How global trends in textile usage threaten freshwater environments. Sci. Total Environ..

[54] Kothari V.K. (2008). 14–Polyester and polyamide fibres—Apparel applications. Polyesters and Polyamides.

[55] Damayanti D., Wulandari L.A., Bagaskoro A., Rianjanu A., Wu H. (2021). Possibility Routes for Textile Recycling Technology. Polymers.

[56] Sahoo S.K., Dash A.K. (2023). 14–Recycled fibres from polyester and nylon waste. Sustainable Fibres for Fashion and Textile Manufacturing.

[57] Production of Elastane Fibres Worldwide from 2017 to 2022; Global Elastane Fibre Production 2022|Statista. https://textileexchange.org/app/uploads/2021/02/Global-Recycled-Standard-v4.0.pdf.

[58] Senthilkumar M. (2011). Elastane fabrics–A tool for stretch applications in sports. Indian J. Fibre Text. Res..

[59] Uyanik S., Kaynak K.H. (2019). Strength, fatigue and bagging properties of plated plain knitted fabrics containing different rates of elastane. Int. J. Cloth. Sci. Technol..

[60] Abdessalem S.B., Abdelkader Y.B., Mokhtar S., Elmarzougui S. (2009). Influence of Elastane Consumption on Plated Plain Knitted Fabric Characteristics. J. Eng. Fibres Fabr..

[61] Korley L.T.J., Epps T.H., Helms B.A., Ryan A.J. (2021). Toward polymer upcycling-adding value and tackling circularity. Science.

[62] Tollini F., Brivio L., Innocenti P., Sponchioni M., Moscatelli D. (2022). Influence of the catalytic system on the methanolysis of polyethylene terephthalate at mild conditions: A systematic investigation. Chem. Eng. Sci..

[63] Li S., Jia L., Shan G., Xiao Y., Liu R. (2022). Preparation of Structural Colors on White Polyester Fabrics without Adding Any Black Additive. Fibers Polym..

[64] Barker R.H. (1975). Additives in Fibers and Fabrics. Environ. Health Perspect..

[65] Juanga-Labayen J.P., Labayen I.V., Yuan Q. (2022). A Review on Textile Recycling Practices and Challenges. Textiles.

[66] Lewis D.M. (2011). 9–The chemistry of reactive dyes and their application processes. Handbook of Textile and Industrial Dyeing.

[67] Clark M. (2011). 1–Fundamental principles of dyeing. Handbook of Textile and Industrial Dyeing.

[68] Ketema A., Worku A. (2020). Review on Intermolecular Forces between Dyes Used for Polyester Dyeing and Polyester Fiber. J. Chem..

[69] Wang J., Cheng W., Gao Y., Zhu L., Pei L. (2019). Mechanism of Accelerant on Disperse Dyeing for PET Fiber in the Silicone Solvent Dyeing System. Polymers.

[70] Apparel and Footwear International RSL Management (AFIRM) Group, Restricted Substances List, 2024 AFIRM RSL. https://afirm-group.com/afirm-rsl/.

[71] Shukla A., Sharma V., Basak S., Ali S.W. (2019). Sodium lignin sulfonate: A bio-macromolecule for making fire retardant cotton fabric. Cellulose.

[72] Sharma V., Wazed Ali S. (2022). A greener approach to impart multiple functionalities on polyester fabric using Schiff base of vanillin and benzylamine. Sustain. Chem. Pharm..

[73] Wojnowska-Baryła I., Bernat K., Zaborowska M., Kulikowska D. (2024). The Growing Problem of Textile Waste Generation–The Current State of Textile Waste Management. Energies.

[74] Chen X., Memon H.A., Wang Y., Marriam I., Tebyetekerwa M. (2021). Circular Economy and Sustainability of the Clothing and Textile Industry. Mater. Circ. Econ..

[75] Nørup N., Pihl K., Damgaard A., Scheutz C. (2018). Development and testing of a sorting and quality assessment method for textile waste. Waste Manag..

[76] Bergfjord C., Holst B. (2010). A procedure for identifying textile bast fibres using microscopy: Flax, nettle/ramie, hemp and jute. Ultramicroscopy.

[77] Zhong R.Y., Xu X., Klotz E., Newman S.T. (2017). Intelligent Manufacturing in the Context of Industry 4.0: A Review. Engineering.

[78] Wu D., Greer M.J., Rosen D.W., Schaefer D. (2013). Cloud manufacturing: Strategic vision and state-of-the-art. J. Manuf. Syst..

[79] Lopes S., Machado S. (2019). IoT Based Automatic Waste Segregator.

[80] Riba J., Cantero R., Canals T., Puig R. (2020). Circular economy of post-consumer textile waste: Classification through infrared spectroscopy. J. Clean. Prod..

[81] Zhou J., Yu L., Ding Q., Wang R. (2019). Textile Fiber Identification Using Near-Infrared Spectroscopy and Pattern Recognition. AUTEX Res. J..

[82] Fibersort, a Circular Solution-YouTube. https://www.youtube.com/watch?v=2zDeRIOzdZo.

[83] Choi S., Choi H. (2019). Eco-friendly, Expeditious Depolymerization of PET in the Blend Fabrics by Using a Bio-based Deep Eutectic Solvent under Microwave Irradiation for Composition Identification. Fibers Polym..

[84] Rosson L., Wang X., Byrne N. (2024). Investigating how the dye colour is impacted when chemically separating polyester-cotton blends. J. Text. Inst..

[85] Peterson A., Wallinder J., Bengtsson J., Idström A., Bialik M., Jedvert K., de la Motte H. (2022). Chemical Recycling of a Textile Blend from Polyester and Viscose, Part I: Process Description, Characterization, and Utilization of the Recycled Cellulose. Sustainability.

[86] Yousef S., Tatariants M., Tichonovas M., Kliucininkas L., Lukošiūtė S., Yan L. (2020). Sustainable green technology for recovery of cotton fibres and polyester from textile waste. J. Clean. Prod..

[87] Li Z. (2020). Separation of waste cotton-polyester blended fabric. IOP Conf. Ser. Mater. Sci. Eng..

[88] Li Z. (2020). Reconstruction of cotton-polyester blended fabric. IOP Conf. Ser. Mater. Sci. Eng..

[89] Loo S., Yu E., Hu X. (2023). Tackling critical challenges in textile circularity: A review on strategies for recycling cellulose and polyester from blended fabrics. J. Environ. Chem. Eng..

[90] Biswas T., Yu J., Nierstrasz V. (2021). Effective Pretreatment Routes of Polyethylene Terephthalate Fabric for Digital Inkjet Printing of Enzyme. Adv. Mater. Interfaces.

[91] Mehmood T., Kaynak A., Dai X.J., Kouzani A., Magniez K., Rubin de Celis D., Hurren C.J., du Plessis J. (2014). Study of oxygen plasma pre-treatment of polyester fabric for improved polypyrrole adhesion. Mater. Chem. Phys..

[92] Fei X., Freeman H.S., Hinks D. (2020). Toward closed loop recycling of polyester fabric: Step 1. decolorization using sodium formaldehyde sulfoxylate. J. Clean. Prod..

[93] Maryan A.S., Montazer M., Damerchely R. (2015). Discoloration of denim garment with colour free effluent using montmorillonite based nano clay and enzymes: Nano bio-treatment on denim garment. J. Clean. Prod..

[94] Huang X., Tan Y., Huang J., Zhu G., Yin R., Tao X., Tian X. (2024). Industrialization of open- and closed-loop waste textile recycling towards sustainability: A review. J. Clean. Prod..

[95] Chen Z., Sun H., Kong W., Chen L., Zuo W. (2023). Closed-loop utilization of polyester in the textile industry. Green Chem. Int. J. Green Chem. Resour. GC.

[96] Mu B., Shao Y., McBride L., Hidalgo H., Yang Y. (2023). Rapid fiber-to-fiber recycling of poly (ethylene terephthalate) and its dye from waste textiles without damaging their chemical structures. Resour. Conserv. Recycl..

[97] Mu B., Yang Y. (2022). Complete separation of colorants from polymeric materials for cost-effective recycling of waste textiles. Chem. Eng. J..

[98] Li Y., Li M., Lu J., Li X., Ge M. (2018). Decoloration of waste PET alcoholysis liquid by an electrochemical method. Water Sci. Technol..

[99] Padmanaban A., Murugadoss G., Venkatesh N., Hazra S., Rajesh Kumar M., Tamilselvi R., Sakthivel P. (2021). Electrochemical determination of harmful catechol and rapid decolorization of textile dyes using ceria and tin doped ZnO nanoparticles. J. Environ. Chem. Eng..

[100] Huang J., Yan D., Dong H., Li F., Lu X., Xin J. (2021). Removal of trace amount impurities in glycolytic monomer of polyethylene terephthalate by recrystallization. J. Environ. Chem. Eng..

[101] Yang Y., Sharma S., Di Bernardo C., Rossi E., Lima R., Kamounah F.S., Poderyte M., Enemark-Rasmussen K., Ciancaleoni G., Lee J. (2023). Catalytic Fabric Recycling: Glycolysis of Blended PET with Carbon Dioxide and Ammonia. ACS Sustain. Chem. Eng..

[102] Choudhury K., Tsianou M., Alexandridis P. (2024). Recycling of Blended Fabrics for a Circular Economy of Textiles: Separation of Cotton, Polyester, and Elastane Fibers. Sustainability.

[103] Guo Z., Adolfsson E., Tam P.L. (2021). Nanostructured micro particles as a low-cost and sustainable catalyst in the recycling of PET fibre waste by the glycolysis method. Waste Manag..

[104] Schmidt A., Watson D., Roos S., Askham C., Poulsen P.B. *Gaining Benefits from Discarded Textiles*; TemaNord; Nordisk Ministerråd: Copenhagen, Denmark, 2016; Gaining Benefits from Discarded Textiles—LCA of Different Treatment Pathways. https://norden.diva-portal.org.

[105] Dukovska-Popovska I., Kjellsdotter Ivert L., Jónsdóttir H., Carin Dreyer H., Kaipia R. (2023). The supply and demand balance of recyclable textiles in the Nordic countries. Waste Manag..

[106] Islam M.T., Rahman M.M., Mazumder N. (2020). Polymers for Textile Production. Frontiers of Textile Materials.

[107] Leonas K. (2023). Fiber-to-Fiber Textile Recycling. Text. World.

[108] Omatsuzawa A. (2023). Textile Technology for Sustainability. Seni Gakkaishi.

[109] Geyer B., Lorenz G., Kandelbauer A. (2016). Recycling of poly(ethylene terephthalate)—A review focusing on chemical methods. Express Polym. Lett..

[110] Al-Sabagh A.M., Yehia F.Z., Eshaq G., Rabie A.M., ElMetwally A.E. (2016). Greener routes for recycling of polyethylene terephthalate. Egypt. J. Pet..

[111] Shojaei B., Abtahi M., Najafi M. (2020). Chemical recycling of PET: A stepping-stone toward sustainability. Polym. Adv. Technol..

[112] Barnard E., Rubio Arias J.J., Thielemans W. (2021). Chemolytic depolymerisation of PET: A review. Green Chem..

[113] Aguado A., Martínez L., Becerra L., Arieta-araunabeña M., Arnaiz S., Asueta A., Robertson I. (2014). Chemical depolymerisation of PET complex waste: Hydrolysis vs. glycolysis. J. Mater. Cycles Waste Manag..

[114] Ghosal K., Nayak C. (2022). Recent advances in chemical recycling of polyethylene terephthalate waste into value added products for sustainable coating solutions—Hope vs. hype. Mater. Adv..

[115] Sun H., Chen Z., Zhou J., Chen L., Zuo W. (2024). Recovery of high-quality terephthalic acid from waste polyester textiles via a neutral hydrolysis method. J. Environ. Chem. Eng..

[116] Costa C., Viana A., Silva C., Marques E.F., Azoia N.G. (2022). Recycling of textile wastes, by acid hydrolysis, into new cellulosic raw materials. Waste Manag..

[117] Peng Y., Yang J., Deng C., Deng J., Shen L., Fu Y. (2023). Acetolysis of waste polyethylene terephthalate for upcycling and life-cycle assessment study. Nat. Commun..

[118] Villar L., Pita M., González B., Sánchez P.B. (2024). Hydrolytic-Assisted Fractionation of Textile Waste Containing Cotton and Polyester. Fibers Polym..

[119] Muszyński M., Nowicki J., Zygadło M., Dudek G. (2023). Comparison of Catalyst Effectiveness in Different Chemical Depolymerization Methods of Poly (ethylene terephthalate). Molecules.

[120] Karayannidis G.P., Chatziavgoustis A.P., Achilias D.S. (2002). Poly(ethylene terephthalate) recycling and recovery of pure terephthalic acid by alkaline hydrolysis. Adv. Pol. Tech..

[121] Quartinello F., Vajnhandl S., Volmajer Valh J., Farmer T.J., Vončina B., Lobnik A., Herrero Acero E., Pellis A., Guebitz G.M. (2017). Synergistic chemo-enzymatic hydrolysis of poly(ethylene terephthalate) from textile waste. Microb. Biotechnol..

[122] Ömeroğullari Başyiğit Z. (2018). Effects of chemical and surface modification on mechanical and chemical properties of polyester fabrics. Düzce Üniversitesi Bilim Ve Teknol. Derg..

[123] Chen G.Q., Dong Z.Q. (2012). Alkaline Hydrolysis of Polyester in the Presence of Ionic Liquids. Adv. Mater. Res..

[124] Zeronian S.H., Collins M.J. (1989). Surface Modification of Polyester by Alkaline Treatments. Text. Prog..

[125] Dave J., Kumar R., Srivastava H.C. (1987). Studies on modification of polyester fabrics I: Alkaline hydrolysis. J. Appl. Polym. Sci..

[126] Čorak I., Tarbuk A., Đorđević D., Višić K., Botteri L. (2022). Sustainable Alkaline Hydrolysis of Polyester Fabric at Low Temperature. Materials.

[127] Kallay N., Grancari A.M., Tomi M. (1990). Kinetics of Polyester Fiber Dissolution. Text. Res. J..

[128] Grancarić A.M., Kallay N. (1993). Kinetics of polyester fiber alkaline hydrolysis: Effect of temperature and cationic surfactants. J. Appl. Polym. Sci..

[129] Saravanana M., Dhinakaran M. (2023). Effect of alkaline hydrolysis on low-stress mechanical properties of polyester/cotton blended weft knitted fabrics. Indian J. Fibre Text. Res..

[130] Musale R.M., Shukla S.R. (2017). Weight reduction of polyester fabric using sodium hydroxide solutions with additives cetyltrimethylammonium bromide and (BMIM)Cl. J. Text. Inst..

[131] Cao J., Meng C., Cheng X., Pan X. (2019). Surface alkali deweighting and dyeing of polyester fabric by one-bath and one-step process. Surf. Innov..

[132] Abedsoltan H. (2023). A focused review on recycling and hydrolysis techniques of polyethylene terephthalate. Polym. Eng. Sci..

[133] Chaonan L., Jihua C. (2007). The study of the recovery of highly purified terephthalic acid from alkali weight-reduction wastewater. Int. J. Environ. Pollut..

[134] Achilias D.S., Karayannidis G.P. (2004). The Chemical Recycling of PET in the Framework of Sustainable Development. Water Air Soil Pollut. Focus.

[135] Palme A., Peterson A., de la Motte H., Theliander H., Brelid H. (2017). Development of an efficient route for combined recycling of PET and cotton from mixed fabrics. Text. Cloth. Sustain..

[136] Yang K., Wang M., Wang X., Shan J., Zhang J., Tian G., Yang D., Ma J. (2024). Polyester/Cotton-Blended Textile Waste Fiber Separation and Regeneration *via* a Green Chemistry Approach. ACS Sustain. Chem. Eng..

[137] Andini E., Bhalode P., Gantert E., Sadula S., Vlachos D.G. (2024). Chemical recycling of mixed textile waste. Sci. Adv..

[138] Ribul M., Lanot A., Tommencioni Pisapia C., Purnell P., McQueen-Mason S.J., Baurley S. (2021). Mechanical, chemical, biological: Moving towards closed-loop bio-based recycling in a circular economy of sustainable textiles. J. Clean. Prod..

[139] Gholamzad E., Karimi K., Masoomi M. (2014). Effective conversion of waste polyester–cotton textile to ethanol and recovery of polyester by alkaline pretreatment. Chem. Eng. J..

[140] Jeihanipour A., Karimi K., Niklasson C., Taherzadeh M.J. (2010). A novel process for ethanol or biogas production from cellulose in blended-fibers waste textiles. Waste Manag..

[141] Sarda P., Hanan J.C., Lawrence J.G., Allahkarami M. (2022). Sustainability performance of polyethylene terephthalate, clarifying challenges and opportunities. J. Polym. Sci..

[142] George N., Kurian T. (2014). Recent Developments in the Chemical Recycling of Postconsumer Poly(ethylene terephthalate) Waste. Ind. Eng. Chem. Res..

[143] Barot A.A., Sinha V.K. (2015). Chemical scavenging of post-consumed clothes. Waste Manag..

[144] Tanaka S., Koga M., Kuragano T., Ogawa A., Ogiwara H., Sato K., Nakajima Y. (2024). Depolymerization of Polyester Fibers with Dimethyl Carbonate-Aided Methanolysis. ACS Mater. Au.

[145] Pham D.D., Cho J. (2021). Low-energy catalytic methanolysis of poly(ethyleneterephthalate). Green Chem..

[146] Yang Y., Lu Y., Xiang H., Xu Y., Li Y. (2002). Study on methanolytic depolymerization of PET with supercritical methanol for chemical recycling. Polym. Degrad. Stab..

[147] Han M., Thomas S., Rane A., Kanny K., Abitha V.K., Thomas M.G. (2019). 5–Depolymerization of PET Bottle via Methanolysis and Hydrolysis. Recycling of Polyethylene Terephthalate Bottles.

[148] Kurokawa H., Ohshima M., Sugiyama K., Miura H. (2003). Methanolysis of polyethylene terephthalate (PET) in the presence of aluminium tiisopropoxide catalyst to form dimethyl terephthalate and ethylene glycol. Polym. Degrad. Stab..

[149] Jang J.Y., Sadeghi K., Seo J. (2022). Chain-Extending Modification for Value-Added Recycled PET: A Review. Polym. Rev..

[150] Pudack C., Stepanski M., Fässler P. (2020). PET Recycling—Contributions of Crystallization to Sustainability. Chem. Ing. Technol..

[151] Liu Q., Li R., Fang T. (2015). Investigating and modeling PET methanolysis under supercritical conditions by response surface methodology approach. Chem. Eng. J..

[152] Goto M., Koyamoto H., Kodama A., Hirose T., Nagaoka S., McCoy B.J. (2002). Degradation kinetics of polyethylene terephthalate in supercritical methanol. AIChE J..

[153] Genta M., Goto M., Sasaki M. (2010). Heterogeneous continuous kinetics modeling of PET depolymerization in supercritical methanol. J. Supercrit. Fluids.

[154] De Castro R.E.N., Vidotti G.J., Rubira A.F., Muniz E.C. (2006). Depolymerization of poly(ethylene terephthalate) wastes using ethanol and ethanol/water in supercritical conditions. J. Appl. Polym. Sci..

[155] Zhou X., Fang C., Yu Q., Yang R., Xie L., Cheng Y., Li Y. (2017). Synthesis and characterization of waterborne polyurethane dispersion from glycolyzed products of waste polyethylene terephthalate used as soft and hard segment. Int. J. Adhes. Adhes..

[156] Fang C., Lei W., Zhou X., Yu Q., Cheng Y. (2015). Preparation and characterization of waterborne polyurethane containing PET waste/PPG as soft segment. J. Appl. Polym. Sci..

[157] Muangmeesri S., Baddigam K.R., Navare K., Apostolopoulou-Kalkavoura V., Witthayolankowit K., Håkansson H., Mathew A.P., Acker K.V., Samec J.S.M. (2024). Recycling of Polyesters by Organocatalyzed Methanolysis Depolymerization: Environmental Sustainability Evaluated by Life Cycle Assessment. ACS Sustain. Chem. Eng..

[158] Kumari A., Kumari A. (2018). Chapter 1–Glycolysis. Sweet Biochemistry.

[159] Vaidya U.R., Nadkarni V.M. (1989). Polyester polyols from glycolyzed PET waste: Effect of glycol type on kinetics of polyesterification. J. Appl. Polym. Sci..

[160] Chen J.Y., Ou C.F., Hu Y.C., Lin C.C. (1991). Depolymerization of poly(ethylene terephthalate) resin under pressure. J. Appl. Polym. Sci..

[161] Kratofil K.L., Hrnjak-Murgić Z., Jelenčić J., Andričić B. (2009). Evaluation of Poly(ethylene-terephthalate) Products of Chemical Recycling by Differential Scanning Calorimetry. J. Polym. Environ..

[162] Muringayil Joseph T., Azat S., Ahmadi Z., Moini Jazani O., Esmaeili A., Kianfar E., Haponiuk J., Thomas S. (2024). Polyethylene terephthalate (PET) recycling: A review. Case Stud. Chem. Environ. Eng..

[163] Zou Y., Reddy N., Yang Y. (2011). Reusing polyester/cotton blend fabrics for composites. Composites. Part B Eng..

[164] Ruschel-Soares R., Contin B., Siqueira M.U., Fernandes P.R.B., Soares N.R., Baruque-Ramos J. (2022). Environmental Impacts of Polyester-Cotton Blend Compared to Cotton Fiber in Brazil. Mater. Circ. Econ..

[165] De Silva R., Wang X.A., Byrne N. (2014). Recycling textiles: The use of ionic liquids in the separation of cotton polyester blends. RSC Adv..

[166] Sasikumar U., Mundkur S., Athalye A. (2023). Strategies for Separating and Recycling Textile Blends. Adv. Environ. Waste Manag. Recycl..

[167] Liu L., Yao H., Zhou Q., Yao X., Yan D., Xu J., Lu X. (2022). Recycling of full components of polyester/cotton blends catalyzed by betaine-based deep eutectic solvents. J. Environ. Chem. Eng..

[168] Ma J., Yang K., Wang M., Shan J., Yang D., Tian G. (2024). Advancements in component separation through chemical methods for recycled polyester/cotton blended textiles. Cellulose.

[169] Haslinger S., Hummel M., Anghelescu-Hakala A., Maattanen M., Sixta H. (2019). Upcycling of cotton polyester blended textile waste to new man-made cellulose fibres. Waste Manag..

[170] Shukla S.R., Harad A.M. (2005). Glycolysis of polyethylene terephthalate waste fibers. J. Appl. Polym. Sci..

[171] Al-Sabagh A.M., Yehia F.Z., Eissa A.M.F., Moustafa M.E., Eshaq G., Rabie A.M., ElMetwally A.E. (2014). Glycolysis of Poly(ethylene terephthalate) Catalyzed by the Lewis Base Ionic Liquid (Bmim)(OAc). Ind. Eng. Chem. Res..

[172] Thomas S., Rane A.V., Kanny K., Abitha V.K., Thomas M.G. (2019). Recycling of Polyethylene Terephthalate Bottles.

[173] Heiran R., Ghaderian A., Reghunadhan A., Sedaghati F., Thomas S., Haghighi A.H. (2021). Glycolysis: An efficient route for recycling of end of life polyurethane foams. J. Polym. Res..

[174] Park S.H., Kim S.H. (2014). Poly(ethylene terephthalate) recycling for high value added textiles. Fash. Text..

[175] El Darai T., Ter-Halle A., Blanzat M., Despras G., Sartor V., Bordeau G., Lattes A., Franceschi S., Cassel S., Chouini-Lalanne N. (2024). Chemical recycling of polyester textile wastes: Shifting towards sustainability. Green Chem. Int. J. Green Chem. Resour. GC.

[176] Fan W., Wang Y., Liu R., Zou J., Yu X., Liu Y., Zhi C., Meng J. (2024). Textile production by additive manufacturing and textile waste recycling: A review. Environ. Chem. Lett..

[177] Baloyi R.B., Gbadeyan O.J., Sithole B., Chunilall V. (2024). Recent advances in recycling technologies for waste textile fabrics: A review. Text. Res. J..

[178] Zangana K.H., Fernandez A., Holmes J.D. (2022). Simplified, fast, and efficient microwave assisted chemical recycling of poly (ethylene terephthalate) waste. Mater. Today Commun..

[179] Luo Y., Selvam E., Vlachos D.G., Ierapetritou M. (2023). Economic and Environmental Benefits of Modular Microwave-Assisted Polyethylene Terephthalate Depolymerization. ACS Sustain. Chem. Eng..

[180] Egan J., Wang S., Shen J., Baars O., Moxley G., Salmon S. (2023). Enzymatic textile fiber separation for sustainable waste processing. Resour. Environ. Sustain..

[181] Du S., Valla J.A., Parnas R.S., Bollas G.M. (2016). Conversion of Polyethylene Terephthalate Based Waste Carpet to Benzene-Rich Oils through Thermal, Catalytic, and Catalytic Steam Pyrolysis. ACS Sustain. Chem. Eng..

[182] Özsin G., Pütün A.E. (2018). A comparative study on co-pyrolysis of lignocellulosic biomass with polyethylene terephthalate, polystyrene, and polyvinyl chloride: Synergistic effects and product characteristics. J. Clean. Prod..

[183] Anbarasu M., Vinitha V., Preeyanghaa M., Neppolian B., Sivamurugan V. (2024). Valorization of PES Textile Waste Through Glycolysis and Aminolysis Using Bimetallic ZnO and Carbon Nitride Nanocomposites. J. Clust. Sci..

[184] Shukla S.R., Harad A.M. (2006). Aminolysis of polyethylene terephthalate waste. Polym. Degrad. Stab..

[185] Ghorbantabar S., Ghiass M., Yaghobi N., Bouhendi H. (2021). Investigation of conventional analytical methods for determining conversion of polyethylene terephthalate waste degradation *via* aminolysis process. J. Mater. Cycles Waste Manag..

[186] Popescu V., Muresan A., Constandache O., Lisa G., Muresan E.I., Munteanu C., Sandu I. (2014). Tinctorial Response of Recycled PET Fibers to Chemical Modifications during Saponification and Aminolysis Reactions. Ind. Eng. Chem. Res..

[187] Lorusso E., Feng Y., Schneider J., Kamps L., Parasothy N., Mayer-Gall T., Gutmann J.S., Ali W. (2022). Investigation of aminolysis routes on PET fabrics using different amine-based materials. Nano Sel..

[188] Ohe T., Yoshimura Y. (2012). Reactions of PET Fibers with Ethylenediamine in a Water Solution Containing Surfactants. Sen’i Gakkaishi.

[189] Lorusso E., Ali W., Leniart M., Gebert B., Oberthür M., Gutmann J.S. (2019). Tuning the Density of Zwitterionic Polymer Brushes on PET Fabrics by Aminolysis: Effect on Antifouling Performances. Polymers.

[190] Spychaj T., Fabrycy E., Spychaj S., Kacperski M. (2001). Aminolysis and aminoglycolysis of waste poly (ethylene terephthalate). J. Mater. Cycles Waste Manag..

[191] Hoang C.N., Dang Y.H. (2013). Aminolysis of poly(ethylene terephthalate) waste with ethylenediamine and characterization of α,ω-diamine products. Polym. Degrad. Stab..

[192] Parab Y.S., Pingale N.D., Shukla S.R. (2012). Aminolytic depolymerization of poly (ethylene terephthalate) bottle waste by conventional and microwave irradiation heating. J. Appl. Polym. Sci..

[193] George N., Kurian T. (2016). Sodium Carbonate Catalyzed Aminolytic Degradation of PET. Prog. Rubber Plast. Recycl. Technol..

[194] Arafat H.A., Jijakli K. (2013). Modeling and comparative assessment of municipal solid waste gasification for energy production. Waste Manag..

[195] Cañete Vela I., Maric J., Seemann M. Valorisation of textile waste via steam gasification in a fluidized bed reactor. Proceedings of the 7th International Conference on Sustainable Solid Waste Management.

[196] Wen C., Wu Y., Chen X., Jiang G., Liu D. (2017). The pyrolysis and gasification performances of waste textile under carbon dioxide atmosphere. J. Therm. Anal. Calorim..

[197] Wu Y., Wen C., Chen X., Jiang G., Liu G., Liu D. (2017). Catalytic pyrolysis and gasification of waste textile under carbon dioxide atmosphere with composite Zn-Fe catalyst. Fuel Process. Technol..

[198] Abdelwahab M.A., Chang B.P., Mohanty A.K., Misra M. (2022). Waste valorisation in sustainable engineering materials: Reactive processing of recycled carpets waste with polyamide 6. Polym. Test..

[199] Hu H., Xu Q., Sun L., Zhu R., Gao T., He Y., Ma B., Yu J., Wang X. (2023). 1 Rapid Hydrolysis of Waste and Scrap PA6 Textiles to ε-Caprolactam. ACS Appl. Polym. Mater..

[200] Braun M., Levy A.B., Sifniades S. (1999). Recycling nylon 6 carpet to caprolactam. Polym. Plast. Technol. Eng..

[201] Cribbs D., Ogale A.A. (2003). Hydrolytic Degradation of Nylon 66 Pile Carpet Fibers. Text. Res. J..

[202] Wang P.Y., Qiang T.T., Ren L.F., Wang X.C. (2011). Moderate Hydrolysis of Nylon 6 Fabric with Different Acids. Adv. Mater. Res..

[203] Minor A., Goldhahn R., Rihko-Struckmann L., Sundmacher K. (2023). Chemical Recycling Processes of Nylon 6 to Caprolactam: Review and Techno-Economic Assessment. Chem. Eng. J..

[204] Bockhorn H., Donner S., Gernsbeck M., Hornung A., Hornung U. (2001). Pyrolysis of polyamide 6 under catalytic conditions and its application to reutilization of carpets. J. Anal. Appl. Pyrolysis.

[205] Czernik S., Elam C.C., Evans R.J., Meglen R.R., Moens L., Tatsumoto K. (1998). Catalytic pyrolysis of nylon-6 to recover caprolactam. J. Anal. Appl. Pyrolysis.

[206] Eimontas J., Yousef S., Striūgas N., Abdelnaby M.A. (2021). Catalytic pyrolysis kinetic behaviour and TG-FTIR-GC–MS analysis of waste fishing nets over ZSM-5 zeolite catalyst for caprolactam recovery. Renew. Energy.

[207] Skvorčinskienė R., Striūgas N., Navakas R., Paulauskas R., Zakarauskas K., Vorotinskienė L. (2019). Thermal Analysis of Waste Fishing Nets for Polymer Recovery. Waste Biomass Valor.

[208] Shukla S.R., Harad A.M., Mahato D. (2006). Depolymerization of nylon 6 waste fibers. J. Appl. Polym. Sci..

[209] Mu B., Shao Y., Yu X., McBride L., Hidalgo H., Yang Y. (2024). High-quality separation and recovery of nylon and dyes from waste carpet via non-destructive dissolution and controlled precipitation for sustainable recycling. Sep. Purif. Technol..

[210] Lv F., Wang C., Zhu P., Zhang C. (2015). Isolation and recovery of cellulose from waste nylon/cotton blended fabrics by 1-allyl-3-methylimidazolium chloride. Carbohydr. Polym..

[211] Kalfas G.A. (1998). Mathematical Modeling of the Depolymerization of Polyamide Mixtures–Part I: Kinetic Mechanism and Parametric Studies in Batch Reactors. Polym. React. Eng..

[212] Duch M.W., Allgeier A.M. (2007). Deactivation of nitrile hydrogenation catalysts: New mechanistic insight from a nylon recycle process. Appl. Catalysis. A Gen..

[213] Vonbrül L., Cordin M., Manian A.P., Bechtold T., Pham T. (2024). Solvent blends for selective elastane dissolution and recovery from mixed polyamide fabrics. Resour. Conserv. Recycl..

[214] Phan K., Ügdüler S., Harinck L., Denolf R., Roosen M., O’Rourke G., De Vos D., Van Speybroeck V., De Clerck K., De Meester S. (2023). Analysing the potential of the selective dissolution of elastane from mixed fibre textile waste. Resour. Conserv. Recycl..

[215] Yin Y., Yao D., Wang C., Wang Y. (2014). Removal of spandex from nylon/spandex blended fabrics by selective polymer degradation. Text. Res. J..

[216] Wang L., Huang S., Wang Y. (2022). Recycling of Waste Cotton Textile Containing Elastane Fibers through Dissolution and Regeneration. Membranes.

[217] Villar L., Schlapp-Hackl I., Sánchez P.B., Hummel M. (2024). High-Quality Cellulosic Fibers Engineered from Cotton–Elastane Textile Waste. Biomacromolecules.

[218] Johansen M.B., Donslund B.S., Henriksen M.L., Kristensen S.K., Skrydstrup T. (2023). Selective chemical disassembly of elastane fibres and polyurethane coatings in textiles. Green Chem. Int. J. Green Chem. Resour. GC.

[219] Levi Strauss & Co Sustainable Fibers–The Foundation of Sustainable Apparel. Sustainable Fibers–Levi Strauss & Co: Levi Strauss & Co. https://www.levistrauss.com/sustainability-report/consumption/sustainable-fibers/.

[220] Control Union Global–Global Recycled Standard GRS–Global Recycled Standard–Control Union Global. https://www.controlunion.com/certification-program/grs-global-recycled-standard/.

[221] Australian Packaging Covenant Organisation-Recycled Content Guide. https://documents.packagingcovenant.org.au/public-documents/Recycled%20Content%20Guide#:~:text=Recycled%20content%20is%20the%20proportion%20%28by%20mass-weight%29%20of,from%20the%20waste%20stream%20during%20manufacturing%20%28excluding%20rework%29.

[222] Environmental Labels and Declarations-Self-Declared Environmental Claims (Type II Environmental Labelling).

[223] Lindex–Circular Fashion Circular Fashion. https://about.lindex.com/sustainability/how-we-work/circular-fashion/.

[224] European Parliament: Textiles and Food Waste Reduction–New EU Rules to Support Circular Economy. *Target. News Serv.* Textiles and Food Waste Reduction: New EU Rules to Support Circular Economy | News | European Parliament. https://www.europarl.europa.eu/pdfs/news/expert/2024/2/press_release/20240212IPR17625/20240212IPR17625_en.pdf.

